# PICALM Regulating the Generation of Amyloid β‐Peptide to Promote Anthracycline‐Induced Cardiotoxicity

**DOI:** 10.1002/advs.202401945

**Published:** 2024-06-27

**Authors:** Mengni Bao, Xiumeng Hua, Xiao Chen, Tao An, Han Mo, Zhe Sun, Menghao Tao, Guangxin Yue, Jiangping Song

**Affiliations:** ^1^ Beijing Key Laboratory of Preclinical Research and Evaluation for Cardiovascular Implant Materials Animal Experimental Centre Fuwai Hospital Chinese Academy of Medical Sciences and Peking Union Medical College Beijing 100037 China; ^2^ State Key Laboratory of Cardiovascular Disease Fuwai Hospital Chinese Academy of Medical Sciences and Peking Union Medical College Beijing 10037 China; ^3^ Shenzhen Key Laboratory of Cardiovascular Disease Fuwai Hospital Chinese Academy of Medical Sciences Shenzhen 518057 China; ^4^ Department of Cardiovascular Surgery Fuwai Hospital Chinese Academy of Medical Sciences and Peking Union Medical College Beijing 10037 China; ^5^ The Cardiomyopathy Research Group Fuwai Hospital Chinese Academy of Medical Sciences and Peking Union Medical College Beijing 10037 China; ^6^ Department of Cardiology Fuwai Hospital Chinese Academy of Medical Sciences and Peking Union Medical College Beijing 10037 China

**Keywords:** amyloid β‐peptide, cardiomyocytes, cardiotoxicity

## Abstract

Anthracyclines are chemotherapeutic drugs used to treat solid and hematologic malignancies. However, life‐threatening cardiotoxicity, with cardiac dilation and heart failure, is a drawback. A combination of in vivo for single cell/nucleus RNA sequencing and in vitro approaches is used to elucidate the underlying mechanism. Genetic depletion and pharmacological blocking peptides on *phosphatidylinositol binding clathrin assembly* (PICALM) are used to evaluate the role of *PICALM* in doxorubicin‐induced cardiotoxicity in vivo. Human heart tissue samples are used for verification. Patients with end‐stage heart failure and chemotherapy‐induced cardiotoxicity have thinner cell membranes compared to healthy controls do. Using the doxorubicin‐induced cardiotoxicity mice model, it is possible to replicate the corresponding phenotype in patients. Cellular changes in doxorubicin‐induced cardiotoxicity in mice, especially in cardiomyocytes, are identified using single cell/nucleus RNA sequencing. *Picalm* expression is upregulated only in cardiomyocytes with doxorubicin‐induced cardiotoxicity. Amyloid β‐peptide production is also increased after doxorubicin treatment, which leads to a greater increase in the membrane permeability of cardiomyocytes. Genetic depletion and pharmacological blocking peptides on *Picalm* reduce the generation of amyloid β‐peptide. This alleviates the doxorubicin‐induced cardiotoxicity in vitro and in vivo. In human heart tissue samples of patients with chemotherapy‐induced cardiotoxicity, *PICALM*, and amyloid β‐peptide are elevated as well.

## Graphical abstract

Single‐cell/nucleus transcriptional profiles of cardiac cells from doxorubicin(DOX)‐induced cardiotoxicity show that amyloid β‐peptide is generated in the heart tissue of patients and mice with anthracycline‐induced cardiotoxicity, and its expression is regulated by phosphatidylinositol binding clathrin assembly (PICALM). Genetic depletion and pharmacological blocking peptide on PICALM in vitro and vivo suppress DOX‐induced amyloid β‐peptide generation in cardiomyocytes and necrotic cell death.

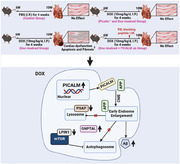

## 1 Introduction

Anthracyclines are cytotoxic antibiotics that are commonly used to treat adult and childhood cancers. For example, doxorubicin (DOX) prolongs the survival of patients with cancer.^[^ ^1^ ^]^ However, the cardiotoxicity of anthracyclines is ≈10% 3.5 months after the final chemotherapy.^[^ ^1^ ^]^ Approximately 98% of patients have experienced cardiotoxicity after the first year of the anthracycline therapy.^[^ ^1^ ^]^ These cardiotoxic side effects limit the cumulative lifetime dose that a patient can receive.^[^ ^2^ ^]^ Current therapeutic strategies for DOX‐induced cardiotoxicity are mostly standard anti‐heart failure and anti‐arrhythmic therapies; however, their effectiveness is limited. To date, specific therapies capable of preventing DOX‐induced cardiotoxicity, especially in heart transplantation are still lacking, because of the mysterious mechanism.^[^ ^3^ ^]^

Impaired mitochondrial quality control processes are associated with mitochondrial dysfunction. This is the major mechanism underlying DOX‐induced cardiotoxicity.^[^ ^4^ ^]^ Preclinical evidence demonstrates that the modulation of mitochondrial quality control can be harnessed for therapeutic benefits against DOX‐induced cardiotoxicity.^[^ ^5^ ^]^ Amyloid β‐peptide (Aβ) is on the mitochondrial membranes, which blocks the transport of nuclear‐encoded mitochondrial proteins to mitochondria, interacts with mitochondrial proteins, disrupts the electron‐transport chain, increases reactive oxygen species (ROS) production, and causes mitochondrial damage.^[^ ^6^ ^]^ Patients with heart failure experience an increase in Aβ levels in their hearts, suggesting that exposure to Aβ peptides impairs the cell viability of cardiomyocytes (CMs).^[^ ^7^ ^]^ However, it is unknown whether higher levels of Aβ peptides were observed during DOX‐induced cardiotoxicity. *PICALM* is the gene encoding phosphatidylinositol binding clathrin assembly (PICALM) protein.^[^ ^8^ ^]^ It plays essential roles in endocytosis, internalization of cell receptors, and intracellular trafficking.^[^ ^9^ ^]^ However, the role of *PICALM* in DOX‐induced cardiotoxicity remains unclear.

Given that increased Aβ peptides in the heart involve cardiac dysfunction,^[^ ^7^, ^10^ ^]^ in this study, we aimed to investigate a possible link among the Aβ peptides, DOX‐induced cardiotoxicity, and the pathogenic effect of *PICALM*. The findings are that *PICALM* mediates the generation of Aβ peptide in CMs on DOX‐induced cardiotoxicity, highlighting the potential of *PICALM* as an effective intervention for cardiotoxicity during anti‐cancer therapy.

## 2 Results

### 2.1 Clinicopathologic Examination of Patients with Cardiotoxicity Caused by Chemotherapy

**Figure 1:**
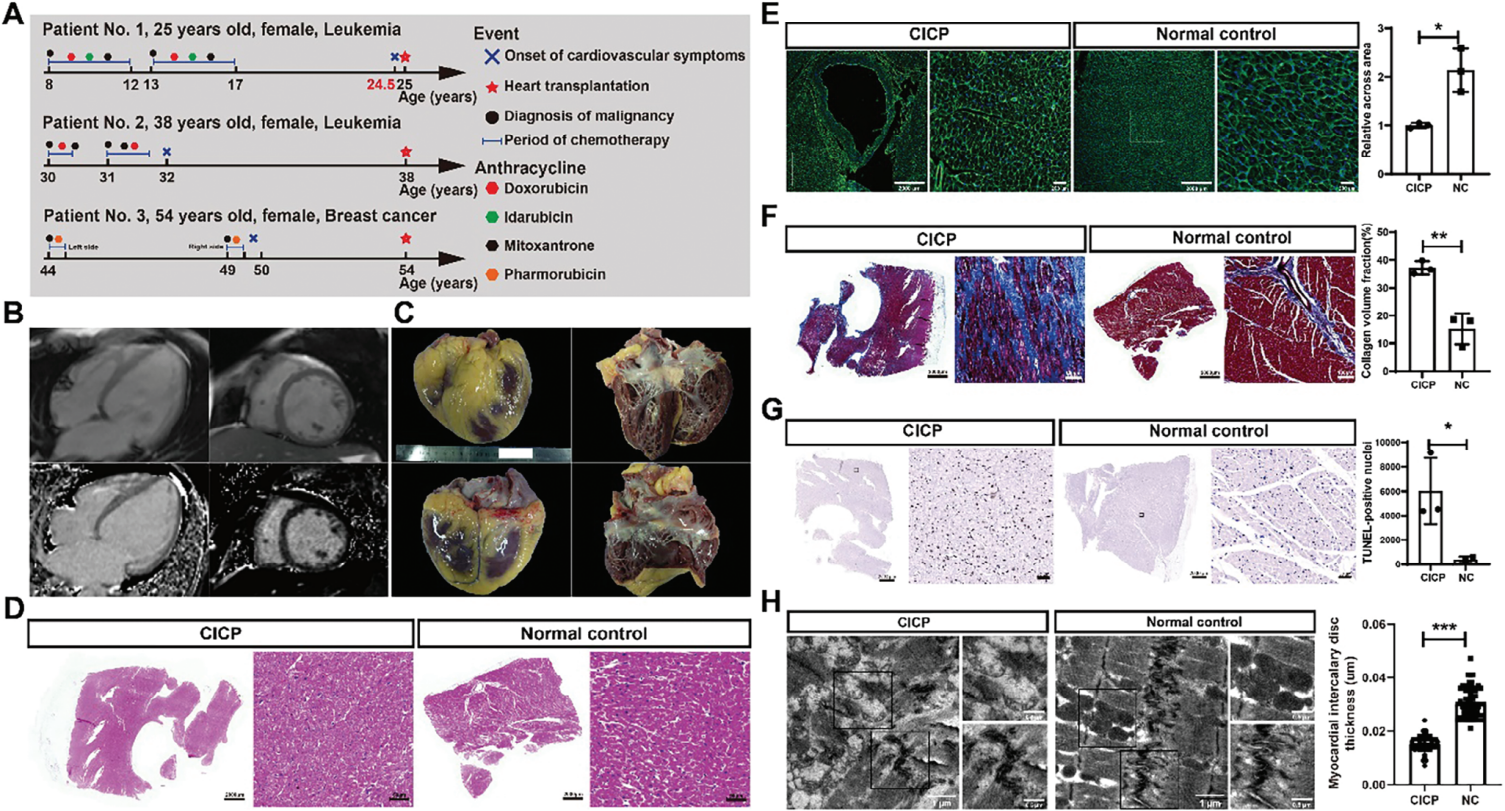
Histological performance of patients with chemotherapy‐induced cardiotoxicity. A) Natural history of the patients, from the diagnosis of malignancy to heart transplantation. B) Representative CMR image of patient No. 1, including LGE. C) Representative graphs of macro‐views of patients with chemotherapy‐induced cardiotoxicity. D) Representative graphs of hematoxylin and eosin (HE) staining of patients with chemotherapy‐induced cardiotoxicity. Scale bars, 2000 µm; scale bars for the magnified images, 50 µm. E–G) Representative and statistical graphs of patients with WGA/Masson/TUNEL‐stained chemotherapy‐induced cardiotoxicity. Scale bars, 2000 µm; scale bars for the magnified WGA/Masson/TUNEL‐stained images, 200/100/50 µm. H) Representative electron micrographs of cardiac muscles derived from the human heart. Scale bars, 1 µm; scale bars for the magnified images, 0.5 µm. NC, normal control; CICP, chemotherapy‐induced cardiotoxicity patient. *n* = 3/group. Statistical analyses were performed using an unpaired t‐test. ^*^ *P* < 0.05; ^**^ *P* < 0.01; ^***^ *P* < 0.001. Data are presented as the mean ± SD.

Chemotherapy‐induced cardiotoxicity is a major concern in anti‐cancer treatment. It may cause severe heart failure.^[^ ^11^ ^]^ To investigate the pathogenesis of chemotherapy‐induced cardiotoxicity, we obtained six human heart samples from the heart transplantation center of Fuwai Hospital, including three patients with chemotherapy‐induced cardiotoxicity and three healthy donors (**Figure** **1**A; Table S1, Supporting Information). All the patients were treated with anthracyclines such as DOX, idarubicin, mitoxantrone, and pharmorubicin (Figure 1A). Interestingly, all the patients received reiterative chemotherapy. We documented the natural history, chemotherapy strategies, and cardiac events from the first symptoms to heart transplantation for each individual as shown in Figure 1A. Patients with end‐stage heart failure and chemotherapy‐induced cardiotoxicity showed evidence of biventricular dilatation in cardiac magnetic resonance (CMR, Figure 1B). Late gadolinium enhancement (LGE) helped identify an enlarged left ventricle, decreased cardiac function, and increased cardiac fibrosis (Figure 1C). Cardiac wasting, fibrosis, and apoptosis are hallmarks of chemotherapy‐related cardiotoxicity.^[^ ^12^ ^]^ In our study, the structure and morphology of CMs were destroyed in patients with chemotherapy‐induced cardiotoxicity (Figure 1D). In patients with chemotherapy‐induced cardiotoxicity, there was a decrease in cardiomyocyte across area, an increase in cardiac fibrosis (Masson: 37.17% vs 15.23%, *P* < 0.01), and an increase in apoptosis (TUNEL‐positive nuclei: 6016.00 vs 361.00, *P* < 0.05) (Figure 1E–G). In contrast to healthy controls, patients who received chemotherapy exhibited cardiac ultrastructural defects, including disrupted myofibrillar networks and mitochondria with disrupted cristae (Figure 1H). Furthermore, the thickness of the myocardial intercalated disc membrane was smaller (0.015 µm) than the thickness of the normal disc (0.030 µm, *P* < 0.001)^[^ ^13^ ^]^ and wide vacuolations were detected in patients with chemotherapy (Figure 1H).

### 2.2 Single‐Nuclei RNA Sequencing (snRNA‐seq) and Single‐Cell RNA sequencing (scRNA‐seq) Revealing the Cardiac Cellular Landscape of DOX‐Induced Cardiotoxicity Mice

**Figure 2:**
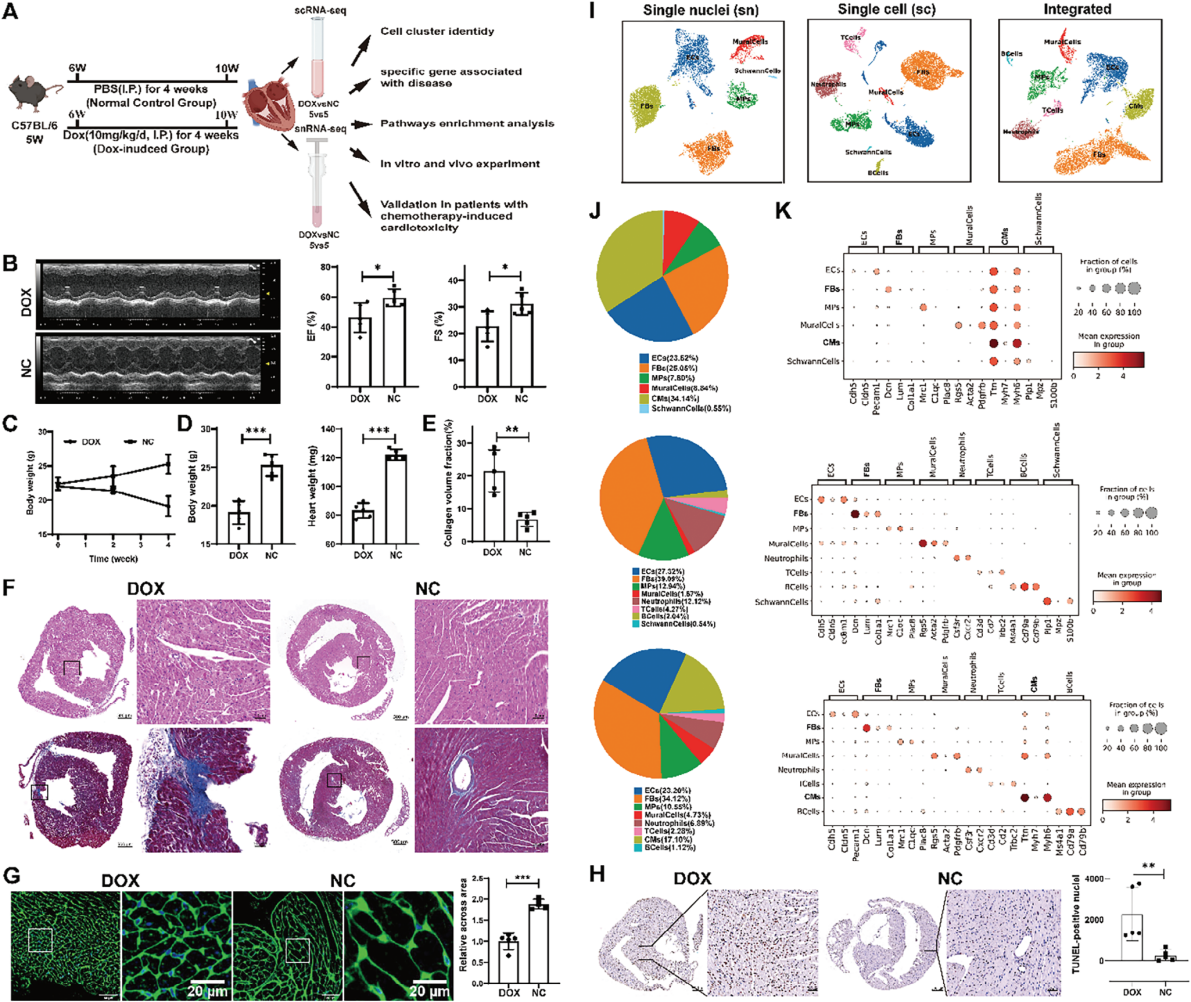
SnRNA‐seq and scRNA‐seq reveal the cellular landscape of DOX‐induced cardiotoxicity. A) Flowchart of our study including the experimental scheme of chronic cardiotoxicity induced by DOX, scRNA‐seq, snRNA‐seq, and analysis for data and experiment. B) Representative images and statistical graphs of cardiac echocardiography of mice injected with phosphate‐buffered saline (PBS) or DOX. EF: ejection fraction; FS: fractional shortening. C) The BW from weeks 6 to 10. D) Body and heart weight of DOX‐treated mice group on week 10. E,F) HE and changes in collagen volume fraction with and without DOX administration. Scan bars, 500 µm; scale bars for the magnified images, 50 µm. G) Representative and statistical graphs of WGA staining in DOX‐treated mice at the tissue level. Scan bars, 50 µm; scale bars for the magnified images, 20 µm. H) Representative and statistical graphs of TUNEL staining in DOX‐treated mice at the tissue level. Scan bars, 500 µm; scale bars for the magnified images, 50 µm. I) UMAP of 18956 nuclei, 24365 cells, and an integrated dataset combining snRNA‐seq and scRNA‐seq data after quality control and data filtering. J,K) Dot plots generated from the integrated dataset displaying characteristic marker genes of each identified cell population, and pie charts showing the proportion of cells within the snRNA‐seq, scRNA‐seq, and integrated datasets. NC, normal control (PBS‐treated mice); DOX, DOX‐treated mice. *n* = 5/group. Statistical analyses were performed using an unpaired t‐test. ^*^ *P* < 0.05; ^**^ *P* < 0.01; ^***^ *P* < 0.001. Data are presented as the mean ± SD.

To define the cardiac cellular and transcriptional landscape of DOX‐induced cardiotoxicity, we established a model of DOX‐treated mice by intraperitoneal injection of DOX (10 mg kg^−1^) weekly for 4 weeks to mimic cardiotoxicity in human patients, following previous reports^[^ ^14^ ^]^ (**Figure** **2**A). DOX‐treated mice experienced a decrease in left ventricular ejection fraction (LVEF: 48.26% vs 59.51%, *P* < 0.05) and a decrease in fractional shortening (FS: 22.74% vs31.19%, *P* < 0.05), indicating the DOX‐induced cardiac functional deterioration, consistent with human clinical data (Figures 1B and 2B). Body weight (BW) decreased more rapidly from 8 to 10 weeks than in the first two weeks in DOX‐treated mice, despite a constant increase in BW in the normal control (NC) group during the 4 weeks post‐DOX injection. This suggests that DOX had a cumulative effect on the target participants (Figure 2C). Compared to the normal group, the final BW and heart weight (HW) at 10 weeks of the DOX‐treated mice group were lower (BW: 19.10 vs 25.28 g, *P* < 0.001; HW: 83.20 vs 122.00 mg, *P* < 0.001; Figure 2D); The area of fibrosis increased by ≈15% in DOX‐treated mice compared to normal group (Figure 2E,F); A significant decreased in across area of CMs of DOX‐treated mice (Figure 2G). The DOX‐treated group showed a significant increase in TUNEL‐positive nuclei (2258 vs 263.6, *P* < 0.01), indicating cardiomyocyte injury (Figure 2H). According to phenotypic features including pathology, the DOX‐induced cardiotoxicity mouse model could mimic the corresponding clinical phenotypes of cardiotoxicity in patients.^[^ ^15^ ^]^

Murine myocardial samples from the DOX‐treated group and normal controls were processed for snRNA‐seq (*n* = 5) or scRNA‐seq (*n* = 5) (**Experimental Section**). Single‐nucleus and single‐cell libraries were sequenced, aligned to the mouse reference genome, and filtered for quality control. Unsupervised clustering, integration, and differential expression analyses were performed using the uniform manifold approximation and projection (UMAP, Figure 2I). After quality control, nuclei samples had average gene and feature counts per cell of 1132 and 2626, respectively, whereas cell counts were 468 and 2025, respectively (Table S2, Supporting Information). The final integrated dataset consisted of 18956 nuclei and 24365 cells representative of 8 major cell types (Figure 2J). Cell identities were validated by expression of cell‐specific marker genes and transcriptional signatures (Figure 2K). Cell types identified in both snRNA‐seq and scRNA‐seq datasets included fibroblasts (FBs, 34.12%), endothelial cells (ECs, 23.20%), CMs (17.10%), mononuclear phagocytes (MPs, 10.55%), neutrophils (6.89%), mural cells (4.73%), T cells (2.28%) and B cells (1.12%) (Figure 2J). A notable benefit of the snRNA‐seq and scRNA‐seq combination was the capacity to obtain not only reads from additional cell types that were not efficiently recovered from enzymatically digested tissues, especially CMs but also to detect more genes and immune cells.

### 2.3 Upregulated *Picalm* Expression in CMs Phenotype Changings During DOX‐Induced Cardiotoxicity

**Figure 3:**
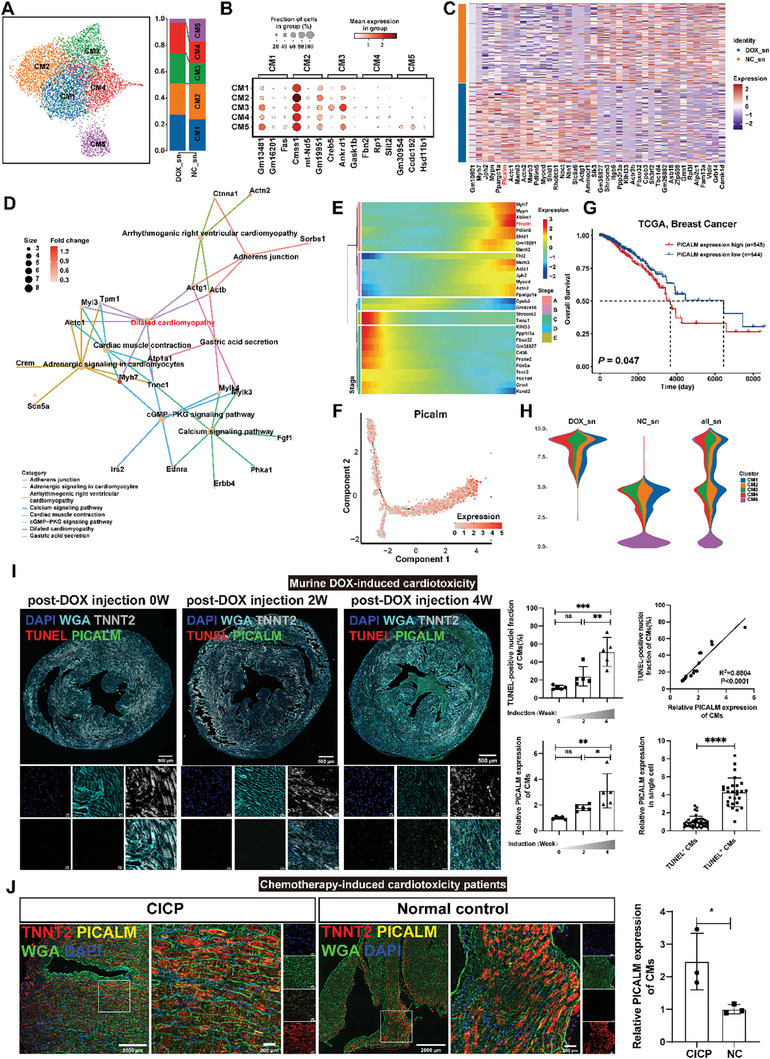
*PICALM* upregulation in CMs of DOX‐treated mice groups and patients with chemotherapy‐induced cardiotoxicity. A) UMAP plot for sub‐cluster analysis of CMs and cardiomyocyte cluster proportions in PBS‐ and DOX‐treated mice. B) Dot plots showing the markers for each cell subcluster. C) Heatmap displaying the top 20 upregulated and downregulated genes ranked by Log2 fold‐change between DOX and NC. D) A heat plot of the associations between upregulated genes and pathways determined by KEGG analysis. Squares indicate that the corresponding gene belongs to the corresponding pathway. E) Heat map showing dynamic changes in gene expression with pseudo‐time. The x‐coordinate from left to right represents the time from small to large, and the y‐coordinate represents the gene. Each point represents the expression of a specific gene at a specified pseudo‐time quantity (mean value). “Cluster” refers to the clustering of genes, clustering genes with similar or similar expression patterns into one cluster. F) Monocle dimension reduction diagram showing the expression of *Picalm*. G) Survival analysis of patients with breast cancer with high or low expression of *PICALM* in tumor samples according to TCGA database. H) Diagram showing the density distribution of different cell types over pseudo‐time. I) OMIHC representative and statistical graphs of PICALM expression in myocardial cells of DOX‐treated mice at different induction times at the tissue level. N > 25 CMs. Scan bars, 500 µm; scale bars for the magnified images, 20 µm. J) OMIHC representative and statistical graphs of PICALM expression in the myocardial cells of patients with chemotherapy‐induced cardiotoxicity. Scan bars, 2000 µm; scale bars for the magnified images, 200 µm. NC, normal control; DOX, DOX‐treated mice; CICP, chemotherapy‐induced cardiotoxicity patients. Mice: *n* = 5/group; human: *n* = 3/group. Student's t‐test was applied to analyze the differences between two groups. Multiple‐group comparisons were made by one‐way ANOVA followed by the Tukey test. ^*^ *P* < 0.05; ^**^ *P* < 0.01; ^***^ *P* < 0.001. Data are presented as the mean ± SD.

6472 CMs were re‐clustered into five clusters using UMAP for further analysis (**Figure** **3**A). CM1‐2 percentage was similar between DOX‐treated mice and normal controls (CM1: 27.4% vs 24.0%; CM2: 23.6% vs 26.9%); CM3‐4 percentage increased in DOX‐treated mice than that in normal controls (CM3: 23.1% vs 16.0%; CM4: 23.0% vs 16.0%), while CM5 percentage decreased in DOX‐treated mice (2.7% vs 17.0%, Figure 3A; Table S3, Supporting Information). Five cardiomyocyte clusters with differing gene expression signatures were identified (Figure 3B; Figure S1, Supporting Information). CMs from DOX‐treated mice exhibited high *Myh7*, *Jph2*, *Mypn*, *Ppargc1a*, *Picalm*, *Actc1*, *Maml3*, and *Msrb3* expression in differentially expressed gene (DEG) analysis (Figure 3C). According to the Kyoto Encyclopedia of Genes and Genomes (KEGG) analysis of upregulated DEG in DOX‐treated mice, DOX greatly affected the cytoskeleton structure and contraction function of CMs, transitioning to the dilated cardiomyopathy (DCM) phenotype, which is consistent with human clinical data (Figure 3D; Table S1, Supporting Information). The KEGG results showed 8 significantly upregulated pathways, including DCM, adrenergic signaling in cardiomyocytes, cardiac muscle contraction, gastric acid secretion, cGMP‐PKG signaling pathway, calcium signaling pathway, adherens junction, and arrhythmogenic right ventricular cardiomyopathy (Figure 3D; Table S1, Supporting Information). Gastric acid secretion, cGMP‐PKG signaling pathway, and calcium signaling pathway were clustered together and were associated with apoptosis and cell death^[^ ^16^ ^]^ (Figure 3D). The results suggest that DOX induces CMs apoptosis, which is consistent with the increased number of apoptotic cells in the DOX group (Figures 1G and 2G).

To explore the temporal relationship between CMs, we calculated the pseudo‐time values for each cardiomyocyte cluster to predict the putative state of cell differentiation using Monocle 2^[^ ^17^ ^]^ (**Experimental Section**). Pseudo‐time trajectory analysis identified three CM branches and five stages (Figure 3E; Figure S2A,B, Supporting Information). Normal CMs were predicted to contain two highly differentiated cell stages (C and E), marked by *Shroom3* and *Cd36* expression (Figure 3E; Figure S2B,C, Supporting Information). In contrast, DOX‐treated mouse samples displayed two highly differentiated cell stages (A and B), marked by *Myh7*, *Picalm*, and *Msrb3* expression (Figure 3E,F; Figure S2C, Supporting Information). It showed convergence toward disease‐associated cardiomyocyte phenotypes in DOX‐treated mice (Figure 3H; Figure S2D, Supporting Information). Pseudo‐time reconstruction analysis of CMs revealed an increase in *Picalm* expression at the end of this trajectory (Figure 3F). According to common cancer (breast cancer, cervical cancer, liver cancer, lung adenocarcinoma, lung squamous cell carcinoma, ovarian cancer, and stomach cancer) survival analysis in The Cancer Genome Atlas (TCGA) database, higher expression levels of *PICALM* in tumor samples indicated shorter survival in breast and stomach cancer (Figure 3G; Figure S3, Supporting Information). To identify this phenotype, the mean expression level of *Picalm* in single cell was upregulated in CM clusters from DOX‐treated mice compared to normal controls (*P* < 0.01) (Figure S4A,B, Supporting Information). Moreover, *Picalm* expression level was higher in CM3 and CM4, whose percentages increased after DOX treatment (Figure S4A, Supporting Information). Consistently, DOX promoted *Picalm* expression, which was 2.79 times higher than controls with a significant difference (*P* < 0.0001, Figure S4C, Supporting Information). Furthermore, *Picalm* expression levels in CMs marked by TNNT2 in DOX‐treated mice were 3.65 times higher than those in controls (Figure S4D, Supporting Information). Immunofluorescence showed *Picalm* expression in the cytoplasm, close to the membrane (Figure S4E, Supporting Information). With DOX treatment (Figure S5, Supporting Information), both the percentage of TUNEL‐positive nuclei and expression level of *Picalm* in CMs marked by TNNT2 increased and correlated positively (Figure 3I). This suggests that increased *Picalm* expression may cause damage to the CM during DOX treatment. Further investigation is necessary to ascertain whether *PICALM* expression increases in patients with chemotherapy‐induced cardiotoxicity. Consistently, *PICALM* expression levels were significantly increased in patients with chemotherapy‐induced cardiotoxicity (Figure S4F, Supporting Information). The opal multicolor immunohistochemistry (OMIHC) results showed that *PICALM* expression in the CMs of patients with chemotherapy‐induced cardiotoxicity increased by approximately two times (Figure 3J). Collectively, increased *PICALM* expression levels in chemotherapy‐induced cardiotoxicity in mouse and human samples were confirmed. These results indicated convergence toward disease‐associated cardiomyocyte phenotypes in DOX‐induced cardiotoxicity with upregulated expression of *PICALM*.

Moreover, the scRNA‐seq datasets showed that DOX did not induce the expression of *Picalm* mRNA in FBs, ECs, MPs, neutrophils, mural cells, T cells, Schwann cells, or B cells (Figure S6, Supporting Information). These results imply that DOX may play certain roles specific to CMs.

### 2.4 Amyloid β (1‐40) Peptide Upregulation in CMs of DOX‐Treated Mice Groups and Patients with Chemotherapy‐Induced Cardiotoxicity

**Figure 4:**
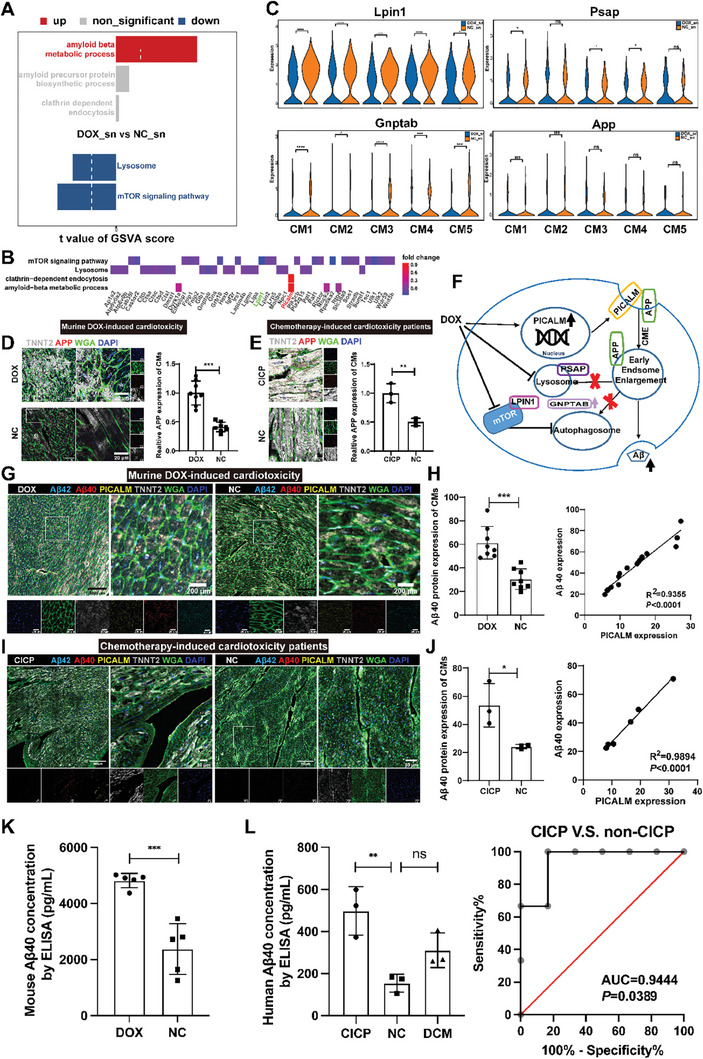
Amyloid β metabolic process pathway and related genes were the major issue in DOX‐induced cardiotoxicity mice and patients with chemotherapy‐induced cardiotoxicity. A) Bar plot for scGSVA analysis of CMs. B) Heat plot of the associations between genes and pathways. C) Violin plots showing the expression of *Lpin1*/*Psap*/*Gnptab*/*App* in each cardiomyocyte cluster. D,E) Representative and statistical graphs of APP expression at the myocardial cells in DOX‐induced cardiotoxicity group and patients. Scan bars, 50 µm; scale bars for the magnified images, 20 µm. F) Diagram showing the potential mechanisms of DOX induction in CMs. G,H) Representative and statistical graphs of Aβ40, PICALM expression at the CMs marked by TNNT2 in DOX‐induced cardiotoxicity mice, and a diagram showing the correlation of PICALM and Aβ40 in mice CMs. Scan bars, 1000 µm; scale bars for the magnified images, 200 µm. I,J) Representative and statistical graphs of Aβ40, PICALM expression at the CMs marked by TNNT2 in patients with chemotherapy‐induced cardiotoxicity, and a diagram showing the correlation of PICALM and Aβ40 in human CMs. Scan bars, 500 µm; scale bars for the magnified images, 50 µm. K) ELISA analysis of mouse serum Aβ40 level in DOX‐induced cardiotoxicity. L) ELISA analysis of human serum Aβ40 level in chemotherapy‐induced cardiotoxicity and plasma Aβ40 levels could distinguish CICP from non‐CICP (AUC = 0.94, *P* = 0.03). NC, normal control; DOX, DOX‐treated mice; CICP, chemotherapy‐induced cardiotoxicity patients. Mice: *n* = 5/group in RNA‐seq and *n* = 8/group in immunoimaging; human: *n* = 3/group. Student's t‐test was applied to analyze the differences between two groups. Multiple‐group comparisons were made by one‐way ANOVA followed by the Tukey test. ^*^ *P* < 0.05; ^**^ *P* < 0.01; ^***^ *P* < 0.001. Data are presented as the mean ± SD.

Gene set analysis was used to identify the pathways involved in DOX‐treated mice.^[^ ^18^ ^]^ Aβ peptide production pathways such as amyloid β metabolism and amyloid precursor protein biosynthesis were activated in DOX‐treated mice, while degradation of Aβ peptide pathways such as lysosome and mTOR signaling pathways were inhibited in DOX‐treated mice (**Figure** **4**A; Figure S7A,B, Supporting Information). The upregulation of *Picalm* expression was involved in the generation of Aβ peptide and the downregulation of *Lpin1* and *Gnptab* was involved in the degradation of Aβ peptide process (Figure 4B,C; Figure S7C, Supporting Information). Collectively, Aβ generation pathways and related genes were upregulated in CMs of DOX‐treated mice.

Amyloid precursor protein (*App*) is a transmembrane precursor protein.^[^ ^19^ ^]^ Its levels were higher in both the DOX‐treated mice and patients. It appears from the data that Aβ can be enhanced by anthracyclines therapy (Figure 4A‐E; Figure S7C, Supporting Information). Based on the gene expression levels of Aβ metabolism, we inferred that *Picalm* induced CMs to produce Aβ during DOX treatment. The upregulation of *Picalm* facilitates clathrin‐dependent endocytosis, which results in early endosomal enlargement. The mTOR‐mediated autophagy defect and lysosome dysfunction aggravated early endosomal enlargement and increased Aβ production (Figure 4F).

Aβ has two forms, amyloid β (1‐40, Aβ40) peptide and amyloid β (1‐42, Aβ42) peptide. Both of which contribute into the pathogenesis of Alzheimer's disease (AD) and cardiovascular disease.^[^ ^20^ ^]^ To elucidate which amyloid β‐peptide was the major form in DOX‐induced cardiotoxicity, we analyzed the expression of Aβ40 and Aβ42 in tissues and serum. The results showed that Aβ and PICALM colocalize by OMIHC staining in both murine models and patients. In DOX‐treated mice, the PICALM, Aβ40, and Aβ42 expression levels in CMs were more increased than the normal controls (Figure 4G,H; Figure S7D, Supporting Information). The expression level of PICALM correlated positively with Aβ40 (R^2^ = 0.9355, *P* < 0.0001, Figure 4H) and Aβ42 (R^2^ = 0.7184, *P* < 0.0001, Figure S7D, Supporting Information). The correlation coefficient between PICALM and Aβ40 (R^2^ = 0.9355) was higher than that between PICALM and Aβ42 (R^2^ = 0.7184). The data suggest that Aβ40 rather than Aβ42 may be the main target affected by *Picalm*. The line profile showed that more than half of the signals from Aβ on the cells and the PICALM signals were co‐located (Figure S7E, Supporting Information). In the patients with chemotherapy‐induced cardiotoxicity, Aβ40 was also highly expressed in CMs in patients, while Aβ42 was similar in two groups (Figure 4I,J; Figure S7F, Supporting Information). It indicates that Aβ40 is the major form in patients with chemotherapy‐induced cardiotoxicity. In addition, the expression levels of Aβ40 in CMs were positively correlated with the expression levels of *PICALM* in human heart samples (R^2^ = 0.9894, *P* < 0.0001, Figure 4J). The Aβ42 expression was also correlated positively with *PICALM* (R^2^ = 0.9375, *P* = 0.0015, Figure S7F, Supporting Information). Further, the concentration of Aβ40 in DOX‐treated mouse serum was increased than in normal mice according to the enzyme‐linked immunosorbent assay (ELISA) experiments (Figure 4K). The corresponding ELISA experiment was conducted with the serum obtained from patients with chemotherapy‐induced cardiotoxicity. The amounts of Aβ40 in the serum of patients with chemotherapy‐induced cardiotoxicity were higher than those in healthy controls (Figure 4L; Table S1, Supporting Information). Moreover, the serum Aβ40 quantity was able to discriminate between cardiotoxicity and non‐cardiotoxicity populations including healthy participants and DCM patients with an area under curve (AUC) of 0.94 (95% CI: 0.79‐1.00, Figure 4L; Figure S7G, Supporting Information). Collectively, *Picalm* can regulate the generation of Aβ40 peptide in CMs during anthracycline therapy. It may lead to cardiotoxicity. The serum Aβ40 peptide is a biomarker for cardiotoxicity in patients.

### 2.5 *Picalm* Regulating the Generation of Aβ Peptide in CMs

**Figure 5:**
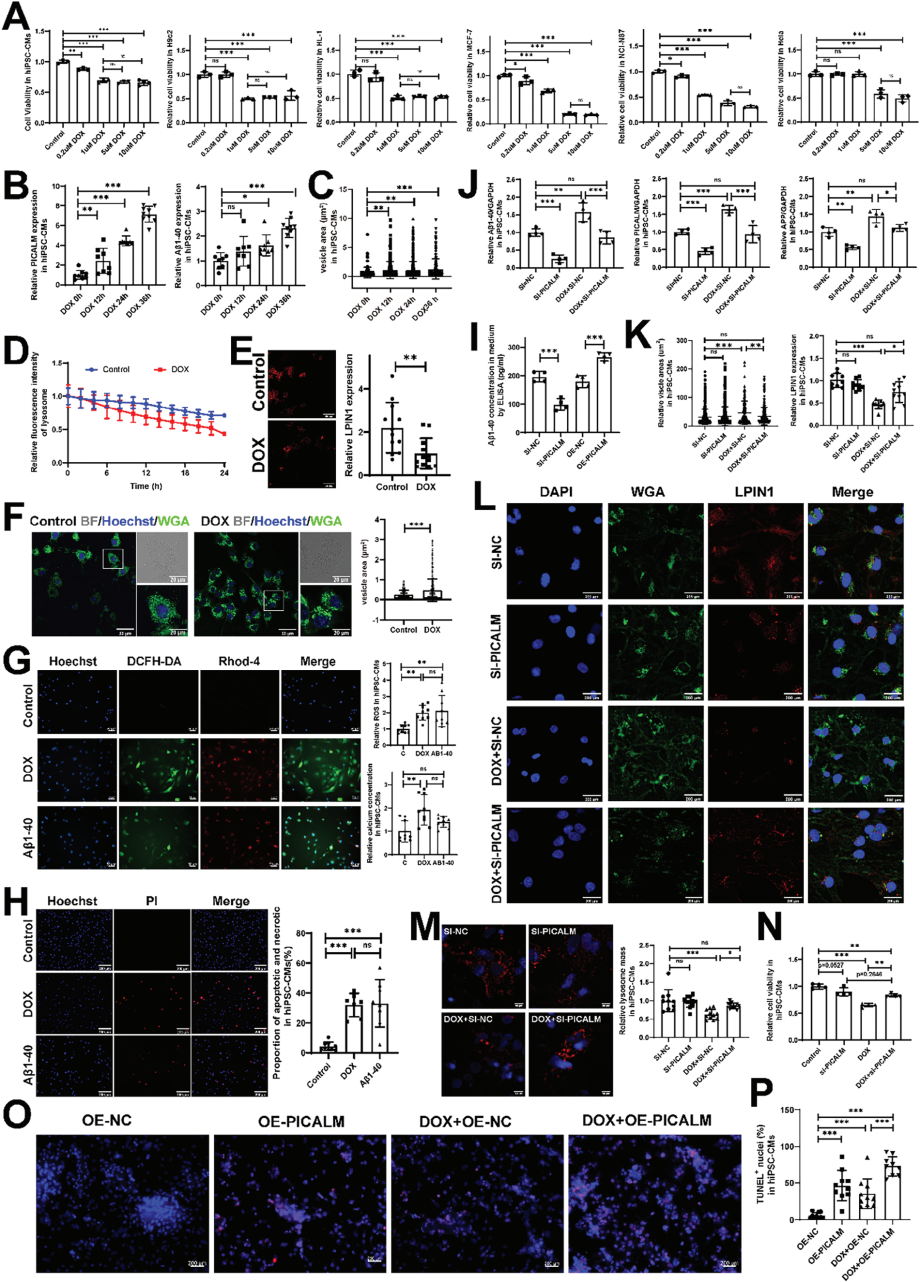
Si‐Picalm can alleviate DOX‐induced hiPSC‐CMs damage and Aβ peptide production. A) Cell viability in different groups. Cells were treated with the indicated concentration of DOX (0–10 µm). After incubation, cell viability was measured by CCK8 assay. B,C) Statistical analysis of vesicle area, PICALM and Aβ40 level of hiPSC‐CMs treated with 1 µM DOX for 0, 12, 24, and 36 h with confocal microscope (Zeiss) by using ImageJ plugins.^[^ ^23^ ^]^ D) The statistical graphs determined using an IncuCyte imaging system. H9c2 cells pre‐stained with 0.5 µM LysoTracker Red for 30 min were treated with 1 µm DOX for 24 h. E) The statistical graphs of LPIN1 expression determined by confocal. F) The WGA staining of DOX‐treated cells and statistical graphs of vesicles area. G) Representative images of ROS and calcium concentration of hiPSC‐CMs treated with DOX or Aβ40 and statistical analysis. Scan bars, 50 µm. H) Representative images of propidium iodide (PI)‐staining of hiPSC‐CMs treated with DOX or Aβ40 and statistical analysis of apoptosis and necrotic cell percentage. Scan bars, 200 µm. I) Statistical analysis of Aβ40 level of hiPSC‐CMs using ELISA in the medium. J) The western blot of the hiPSC‐CMs groups including SI‐NC, SI‐PICALM, DOX+SI‐NC, DOX+SI‐PICALM, and statistical graphs of PICALM, APP, and Aβ40 expression. K,L) Representative images and statistical analysis of WGA/LPIN1 staining in the hiPSC‐CMs groups. Scan bars, 200 µm. M) Representative images and statistical analysis of lysosome tracking in the hiPSC‐CMs groups. Scan bars, 50 µm. N) The cell viability in groups. O,P) Representative images and statistical analysis of TUNEL assay in the hiPSC‐CMs groups. Scan bars, 200 µm. DOX, DOX‐treated cells; SI‐NC, SI‐GFP; OE‐NC, overexpression vector. Student's t‐test was applied to analyze the differences between two groups. Multiple‐group comparisons were made by one‐way ANOVA followed by the Tukey test. ^*^ *P* < 0.05; ^**^ *P* < 0.01; ^***^ *P* < 0.001. Data are presented as the mean ± SD.

A cell model induced by DOX was built to verify this hypothesis and further elucidate the mechanism (Figure 4F). To recapitulate the many features of human CM physiology, a human induced pluripotent stem cell‐derived cardiomyocytes (hiPSC‐CMs)‐based model of anthracycline‐induced cardiotoxicity was included (Figure S8, Supporting Information). In the first step, cells were treated with DOX at different concentrations. We found that 1 µm DOX could reduce the viability of cardiomyocyte cells (hiPSC‐CMs/H9c2/HL‐1). However, this concentration of DOX had no significant effect on the viability of non‐cardiomyocyte cells such as mouse embryo fibroblasts (NIH3T3), rat cardiac fibroblasts (RCFs), human umbilical vein endothelial cells (HUVECs), and a macrophages line (Raw246.7). These data confirmed the effects of DOX on CMs (**Figure** **5**A; Figure S9A, Supporting Information). Additionally, 1 µM DOX could significantly reduce the activity of a breast cancer cell line (MCF‐7) and a stomach cell line (NCI‐N87); however, not on a cervical cancer cell line (HeLa) (Figure 5A). These results are consistent with the efficacy of DOX in clinical practice, where DOX has been shown to be more effective against breast and stomach cancers than cervical cancers.^[^ ^21^ ^]^ Then, we monitored how treatment with 1 µM DOX affected cell living and proliferation. IncuCyte microscopic images showed that treatment with DOX reduced cell proliferation to the midpoint of the treatment period and affected the number of cells to reach the LogIC50 (Figure S9B, Supporting Information).

To monitor the impact of DOX treatment on the production of PICALM and Aβ40 peptide, we selected four timepoints. We found that induction of DOX resulted in increased vesicle area, higher expression of PICALM, and higher expression of Aβ40 using confocal microscope (Figure 5B,C; Figure S9C, Supporting Information). These results further confirmed that DOX could induce the expression of PICALM and generation of Aβ40. In terms of signaling pathways (Figure 4F), DOX promoted lysosome mass reduction (Figure 5D; Figure S9D, Supporting Information),^[^ ^22^ ^]^ *LPIN1* expression level decreased (Figure 5E), and vesicle area increased (Figure 5F). The data suggests that DOX inhibits lysosome and autophagy pathways, resulting in vesicles secreting more Aβ40. We compared the effects of Aβ40 and DOX on CMs to better understand the impact of Aβ40 on DOX‐induced cardiotoxicity. DOX and Aβ40 led to an increase in ROS and calcium ions (Figure 5G; Figure S10A, Supporting Information), in which free radicals can destroy the cytoskeleton (Figure S10B, Supporting Information), cell membrane functions (Figure S10C, Supporting Information, and organelle functions (Figure 5F; Figure S10D, Supporting Information), and promote an increase in membrane permeability and cause cell death (Figure 5H).

To examine the effect of PICALM on Aβ40 peptide production, we knocked down and overexpressed *Picalm* in hiPSC‐CMs. The expression of the Aβ40 peptide was assessed using ELISA (Figure S10E, Supporting Information). *PICALM* knockdown resulted in a decrease in Aβ40 levels in cellular supernatant, while overexpression of *PICALM* resulted in increasing in Aβ40 levels (Figure 5I). To test whether PICALM knockdown could salvage DOX‐induced cell damage, knockdowns of the *PICALM* in hiPSC‐CMs and H9c2 cells were performed and then treated with DOX. The PICALM and Aβ peptide expression in the DOX group were compared with the control group. The si‐*Picalm* significantly reduced the DOX‐induced Aβ40 peptide production, PICALM expression, and APP expression (Figure 5J; Figure S10E–I, Supporting Information). We then analyzed the effect of DOX on the basis of knockout of *PICALM* on the lysosome and autophagy signaling pathways, and found that inhibition of *Picalm* expression could alleviate DOX‐induced dysfunction of the lysosome, autophagy, and organelles (Figure 5K–M). Furthermore, the CCK8 assay showed that si‐*Picalm* alleviated DOX‐induced damage (Figure 5N). To investigate whether overexpression of Picalm worsens DOX‐induced cardiotoxicity, hiPSC‐CMs were transfected with plasmids that overexpressed PICALM, followed by DOX induction (Figure S10E, Supporting Information). TUNEL assay showed overexpression of PICALM exacerbated DOX‐induced toxicity (Figure 5O,P). Collectively, *Picalm* can generate Aβ peptide in CMs, and Aβ peptide, in turn, causes cardiotoxicity. In addition, we explored whether *PICALM* knockout affected the effectiveness of DOX in oncology treatment. *PICALM* knockout was performed in MCF‐7 and NCI‐N87, followed by DOX treatment in both tumor cell lines. These results indicate that *PICALM* knockout has no effect on DOX treatment (Figure S11, Supporting Information).

### 2.6 Genetic Depletion of *Picalm* Attenuating DOX‐Induced Cardiotoxicity In Vivo

**Figure 6:**
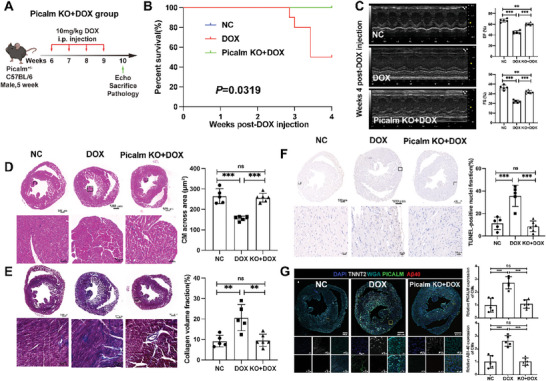
Genetic depletion of PICALM attenuated DOX‐induced cardiotoxicity in vivo. A) The treatment experiment design. B) Survival analysis of the three groups. C) Representative and statistical graphs of EF and FS evaluated by echocardiography of normal control, DOX induction on wild‐type mice and DOX induction of Picalm knockout mice. D,E) Representative and statistical graphs of HE and Masson staining of normal control, DOX induction on wild‐type mice and DOX induction of Picalm knockout mice. F) The IHC images of TUNEL of normal control, DOX induction on wild‐type mice and DOX induction of Picalm knockout mice. G) Representative and statistical graphs of WGA, Aβ40, PICALM expression at the CMs marked by TNNT2 in normal control, DOX induction on wild‐type mice and DOX induction of Picalm knockout mice. NC, normal control, *n* = 5; DOX, DOX‐treated mice, *n* = 5; DOX+KO, DOX‐treated Picalm knockout mice, *n* = 6. Student's t‐test was applied to analyze the differences between two groups. Multiple‐group comparisons were made by one‐way ANOVA followed by the Tukey test. ^*^ *P* < 0.05; ^**^ *P* < 0.01; ^***^ *P* < 0.001. Data are presented as the mean ± SD.

Conventional *Picalm* knock out mice were used to investigate the role of *Picalm* in the pathogenesis of DOX‐induced cardiotoxicity (Figure S12, Supporting Information). Three groups were designed, including wild‐type mice (*n* = 5), wildtype with DOX treatment (*n* = 5), and *Picalm* ^+/−^ mice with DOX treatment (*n* = 6) as shown in **Figure** **6**A. *Picalm* ^+/−^ mice had longer survival time than the wild‐type mice under the same DOX treatment (*P* = 0.0319) (Figure 6B). Genetic depletion of *Picalm* rescued the cardiac functions of DOX‐induced cardiotoxicity, in which EF and FS in *Picalm* ^+/−^ mice increased significantly compared to those in DOX‐treated mice (Figure 6C).

A higher across area of CMs than that in the DOX group, and the lower fibrosis abundant in the heart of the *Picalm* ^+/−^ group confirmed the role of PICALM in DOX‐induced cardiotoxicity (Figure 6D,E). Further, apoptosis of CMs decreased in the *Picalm* ^+/−^ group (Figure 6F). Aβ40 peptide in CM expression also decreased (Figure 6G). Collectively, these data suggest that the genetic depletion of *Picalm* attenuates DOX‐induced cardiotoxicity in vivo.

### 2.7 Anti‐PICALM Antibody Blocking Peptides (BPs) Attenuating DOX‐Induced Myocardial Damage and Decreasing Aβ40 Peptide Production In Vivo

**Figure 7:**
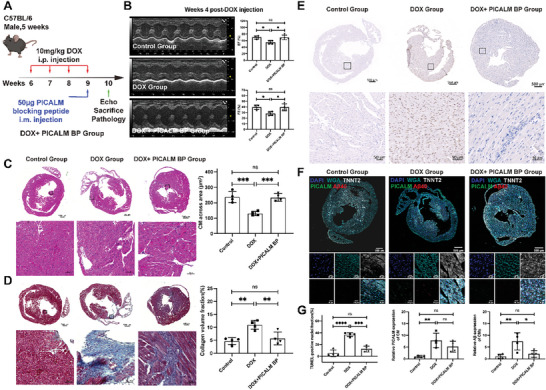
DOX‐induced cardiotoxicity is suppressed by anti‐PICALM antibody BP. A) Treatment experiment design. B) Representative and statistical graphs of EF and FS evaluated by echocardiography in the control, DOX, and DOX + PICALM BP group. C,D) Representative and statistical graphs of HE and Masson staining of the control, DOX, and DOX + PICALM BP group. E) IHC images of TUNEL of the control, DOX, and DOX + PICALM BP group. F) Representative graphs of WGA, Aβ40, PICALM expression at the CMs marked by TNNT2 in the control, DOX, and DOX + PICALM BP group. G) Statistical graphs of TUNEL‐positive cells, and PICALM, Aβ40 expression at the CMs marked by TNNT2 in the control group, DOX, and DOX + PICALM BP group. DOX, DOX‐treated mice. Mice: *n* = 4/group. Student's t‐test was applied to analyze the differences between two groups. Multiple‐group comparisons were made by one‐way ANOVA followed by the Tukey test. ^*^ *P* < 0.05; ^**^ *P* < 0.01; ^***^ *P* < 0.001. Data are presented as the mean ± SD.

Genetic depletion of *Picalm* expression could attenuate DOX‐induced myocardial damage and Aβ peptide production in vitro and in vivo. The animal model has been constructed to investigate the effects of anti‐PICALM antibody (50 µg per mouse) by intramyocardial injection. This led to the development of new clinical applications (**Figure** **7**A). Administration of the anti‐PICALM antibody rescued cardiac function in mice with DOX‐induced cardiotoxicity. EF and FS were noticeably higher in mice treated with the anti‐PICALM antibody than that in those treated with DOX (Figure 7B). Histological analysis revealed a greater CM area than that in the DOX group. Lower levels of fibrosis in the heart of the DOX anti‐PICALM antibody group confirmed the effectiveness of anti‐PICALM antibody therapy (Figure 7C,D). Furthermore, anti‐PICALM antibody decreased Cs apoptosis (Figure 7E,G). PICALM expression decreased in mice treated with the anti‐PICALM antibody (Figure 7F,G). Aβ40 peptide in CM expression also decreased after anti‐PICALM antibody treatment (Figure 7F,G). Collectively, these results suggest that anti‐PICALM antibodies can attenuate the cardiotoxicity caused by DOX and decrease the production of Aβ40 in vivo.

## 3 Discussion

Chemotherapy has been one of the most effective treatment modalities for cancer since its emergence in the 1940s. However, growing concerns have been raised regarding its cardiotoxic effects.^[^ ^24^ ^]^ Owing to limited research on its cellular mechanism, there are no efficient therapeutic strategies available for chemotherapy‐associated cardiotoxicity.^[^ ^25^ ^]^ Understanding the specific cellular regulatory processes of cardiotoxicity under diseased conditions is of fundamental importance for successful drug development, such as for developing drugs for the treatment of DOX‐induced cardiotoxicity.^[^ ^15^ ^]^ In this study, we investigated the pathological characteristics of DOX‐induced cardiotoxicity and established a mouse model of chronic DOX‐induced cardiotoxicity. Cells and nuclei from the cardiac tissue of the mouse model were sequenced using snRNA‐seq and scRNA‐seq. Specific changes in the expression profiles, subpopulation composition, and intercellular communication in cardiac tissues of the mouse model were defined, especially in the CMs. By comparing gene expression profiles from normal participants, we showed the enrichment of markers of CM stress induced‐cardiotoxicity in the hearts. Based on this heterogeneity, we clustered the transcriptionally related cells or genes, each of which was likely related to different cellular functions. In addition, the data showed that the transcriptomic states of CMs in human hearts were continuous rather than discrete. Previous studies on cardiac tissues have reported a change at the tissue level, but not single cell levels. To the best of our knowledge, this is the first report of a single nucleus/cell study of the cardiac tissues of a mouse model of chronic DOX‐induced cardiotoxicity.

Here, we report a significant stimulative effect of *Picalm* on DOX‐induced cardiotoxicity in rodent and cardiomyocytes (hiPSC‐CMs) in vitro and in a DOX‐induced cardiotoxicity mouse model. The data showed an interaction between *Picalm* and Aβ40 peptide, which played an essential role in the induction of cardiomyocyte apoptosis (**TOC**). *Picalm* knockout mice and iPSC‐CM experiments results indicated that *Picalm* could promote the generation of Aβ peptide, and then led to DOX‐induced cardiotoxicity. This intervention efficiently prevented DOX‐induced cardiotoxicity, highlighting the therapeutic value of *Picalm*. In addition, *PICALM* knockdown did not affect the therapeutic effects in breast and stomach cancers in vitro. TCGA data indicated that low PICALM expression was associated with a better prognosis. PICALM‐BP may be a novel treatment for anthracycline‐induced cardiotoxicity in the clinic and does not affect the effectiveness of DOX in tumor treatment. Collectively, PICALM may be a novel target for the treatment of anthracycline‐induced cardiotoxicity in clinical settings. However, drugs targeting *Picalm* are not currently available. Further investigations are required to develop new drugs for clinical use.

Aβ peptide is produced through the proteolytic processing of a transmembrane protein, APP, by β‐ and γ‐secretases. Aβ peptide accumulation in the brain is proposed to be an early toxic event in the pathogenesis of AD, which is the most common form of dementia associated with plaques and tangles in the brain.^[^ ^26^ ^]^ Aβ peptide accumulation has also been found in the skeletal muscles of patients affected by sporadic inclusion‐body myositis and atherosclerotic aortas.^[^ ^27^ ^]^ In addition, the β‐amyloid peptide was observed in heart failure, which contributes to cardiac dysfunction via decreasing the cell viability in both ECs and CMs.^[^ ^7a^ ^]^ There were no reports about roles of regarding the β‐amyloid peptide in DOX‐induced cardiotoxicity, although it was reported that DOX‐treated rats significantly exhibited AD‐like brain injury, increased amyloid burden, enhanced neuroinflammation and apoptosis, and multifocal histological injury in the cerebral cortex with widespread vacuolations.^[^ ^28^ ^]^ This present study is the first to reveal that DOX induces amyloid generation, causing cardiotoxicity. Also, the relationship between Aβ and membrane permeability has been reported. Recent advances indicate that these interactions are a clear picture of the Aβ induced membrane disruption.^[^ ^29^ ^]^ The serum Aβ40 levels could be applied as a biomarker for discriminating cardiotoxicity patients (Figure 4G). This could serve as a diagnostic reference in the absence of an effective diagnostic strategy. This needs a larger cohort to confirm the effectiveness of serum Aβ40 levels as a specific biomarker. Taken together, our data suggest a possible mechanism of DOX‐induced cardiotoxicity. DOX upregulated the expression of *Picalm*, which induces the accumulation of Aβ to induce membrane disruption to result in the disturbance of cellular homeostasis. Combining our findings and previous studies, β‐amyloid peptide participated in the process of cardiovascular disease.^[^ ^30^ ^]^ This may indicate whether there was a connection between the brain and the heart, an emerging and essential topic that requires additional investigation.^[^ ^31^ ^]^

A limitation of our study was the lack of a mechanism by which *Picalm* expression was regulated by DOX. Further investigations on this point should be conducted in the future. In addition, the sample size of human cardiac tissues is rather small because of difficulties in obtaining them. All heart samples in this study were obtained from female patients in this study. Thus, these findings should be further verified in male patients.

In summary, we performed a comprehensive cellular analysis of DOX‐induced cardiotoxicity. This study primarily focused on CMs. We identified *Picalm* as the hallmark gene. It was responsible for the generation of Aβ in CMs, which increased membrane permeability and induced cardiotoxicity in both human tissues and animal models. A potential therapy strategy for patients with end‐stage heart failure caused by chemotherapy could be targeting *Picalm*.

## 4 Experimental Section

### Model Building and Grouping and Picalm Knockout Mice Construction

Five‐week‐old wild‐type male C57BL/6 mice (Vital River Laboratories, Beijing, China) were maintained in a specific pathogen‐free facility and provided with a normal diet and drinking water. And *Picalm*‐KO mice (C57BL/6Smoc‐Picalm^em1Smoc^, Cat. NO. NM‐KO‐200880) were purchased from the Shanghai Model Organisms Center, Inc. This study was approved by the Animal Ethics Committee of the Fuwai Hospital (FW‐2022‐0015). The guidelines of Directive 2010/63/EU of the European Parliament on the protection of animals used for scientific purposes were followed in this animal experiment. The animals were randomly divided into two groups: NC and DOX group. After 1 week of adaptive feeding, the DOX group was administered by weekly intraperitoneal injections of 10 mg kg^−1^ of DOX hydrochloride (Selleck, S1208) for four consecutive weeks. The NC group was intraperitoneally injected with PBS at the same time. The weight of the mice was recorded weekly. After 4 weeks of injection, the mice were examined using echocardiography (VisualSonics, Vevo2100 system).

### hiPSC‐CM Culture

Human CMs were differentiated from human induced pluripotent stem cells based on an established protocol using an activator and an inhibitor of the Wnt signaling pathway sequentially and matured using maturation media supplemented with L‐lactate, L‐ascorbic acid, and recombinant human albumin.^[^ ^32^ ^]^ Human CMs were characterized by immunostaining with TNNT2 antibodies and the percentage of TNNT2^+^ cells was counted and calculated based on the images (Figure S12A, Supporting Information). Over 90% of differentiated cells were TNNT2^+^. Similar results were observed using flow cytometry characterization and ≈97% of cells were TNNT2^+^ (Figure S12B, Supporting Information). These cells are suitable models for mimicking the response of human CMs to chemical or genetic perturbations. Cell differentiation was supported by Beijing Vercelldo Technological Lab (part of Beijing Wosida Xibao Jishu Company Limited).

Cells were grown in growth media (Cor.4U Complete Culture Medium which contains 10% fetal bovine serum (FBS) and other essential nutrients, Catalog number Ax‐M‐HC250 from Ncardia) and treated in minimal media (BMCC Serum‐Free Culture Medium, Catalog Number Ax‐MBMCC250 from Ncardia, supplemented with 1% fetal bovine serum from Thermo Fisher Scientific) in a humidified incubator with 5% CO_2_ and 37 °C. Cryo‐preserved hiPSC‐CMs were thawed in 75‐cm^2^ flasks, cultured in growth media for 3 days, and reseeded into multi‐well plates. Then, hiPSC‐CMs were cultured in minimal media for 1.5 days before drug treatment and maintained in minimal media during drug treatment with media exchanged every other day.

### Cell Culture

H9c2 (2‐1) (ATCC CRL‐1446), non‐cardiomyocyte cells such as NIH3T3, RCF, HUVEC, and Raw246.7, and tumor cells line (MCF‐7 cells, NCI‐N87 cells, and HeLa cells) were cultured in Dulbecco's Modified Eagle Medium (DMEM, Gibco) supplemented with 10% FBS in 5% CO_2_ at 37 °C. HL‐1 (ATCC CRL‐12197) was cultured in Minimum Essential Medium (MEM, Gibco) supplemented with 10% FBS in 5% CO_2_ at 37 °C. When the cell density reached ≈80%, the cells were digested with 0.25% trypsin every two to three days.

### Human Heart Sample Collection

The use of human tissue in this study was approved by the Human Ethics Committee of Fuwai Hospital, Chinese Academy of Medical Sciences (No. 2021‐1465). Written informed consent was obtained from each patient. Human heart samples were collected from patients who underwent heart transplantation in the operating room. The patient who experienced DOX‐induced cardiotoxicity (*n* = 3). Healthy heart samples (*n* = 3) were obtained from brain‐dead donors with a normal circulatory supply who were not suitable for transplantation for technical or noncardiac reasons, such as BW mismatch, according to the guidelines of the China Transplant Services. Detailed information is provided in Table S1 (Supporting Information).

### ScRNA‐seq

Following histological examination, five hearts from each group were pooled to isolate single cells. The tissues were cut into small pieces in PBS and digested with 200 U/ml collagenase type II (Worthington, 43J14367B) in a 37 °C water bath with mild shaking. The single‐cell suspension was filtered with a 40 µm cell strainer and collected by centrifugation at 400 g for 5 min. The supernatant was discarded, and the cell pellets were resuspended in 1 mL DMEM containing 10% FBS.^[^ ^33^ ^]^ Isolated non‐myocytic cells from each heart were counted and used for single‐cell transcriptomic library preparation on the Chromium controller (10X Genomics). Cells were loaded into each channel and processed using the Chromium Single Cell 3′ v1 reagent kit. Following capture and lysis, the synthesis and amplification were conducted for 14 cycles according to the manufacturer's protocol. The amplified cDNA was used to construct Illumina sequencing libraries, each sequenced in one lane of the Illumina HiSeq X ten.

### SnRNA‐seq

Nucleus was extracted from five heart tissues in each group. After removing from −80 °C, the tissue was dissected in cold PBS. The shredded samples were then transferred to the 30 mL lysis solution and stirred for 20 s. The stirred liquid was homogenized in the Dounce tissue grinder set (pestle B) for 10 strokes. The homogenate was passed through 100 and 70 µm filters successively. After centrifugation at 700 g for 10 min at 4 °C, the supernatant was discarded, and the pellet was resuspended using 23 mL preservation solution. The remaining 7 mL of preservation solution was poured into a centrifuge tube, followed by slow pouring of 23 mL of the resuspension into the centrifuge tube and centrifugation for 1 h at 13 000 rpm. After centrifugation, the supernatant was discarded, and the pellet was resuspended using 1% bovine serum albumin.^[^ ^34^ ^]^ The nuclei were counted using Trypan blue and further processed following the manufacturer's protocol for 10x Genomics. The snRNA‐seq libraries were prepared using the Chromium Next GEM Single Cell 3′ GEM, Library & Gel Bead Kit, version 3.1, 16 reactions and sequenced using the Illumina HiSeq X ten.

### Integration of Single‐Cell and Single‐Nuclei Datasets

Scanpy v1.8.2^[^ ^35^ ^]^ was used for quality control, dimensionality reduction, and clustering using Python 3.7. For each sample dataset, the expression matrix was filtered using the following criteria: 1) cells with gene count <200 or with top 2% gene count were excluded; 2) cells with top 2% UMI count were excluded; 3) cells with mitochondrial content > 20% were excluded; 4) genes expressed in less than five cells were excluded. After filtering, 42 764 cells were retained for the downstream analysis, with on average 1274 genes and 3243 UMIs per cell. The raw count matrix was normalized to total counts per cell and logarithmically transformed into normalized data matrix. Top 2000 variable genes were selected by setting flavor = “seurat”. Principle Component Analysis (PCA) was performed on the scaled variable gene matrix, and top 20 principal components were used for clustering and dimensional reduction. Cells were separated into 25 clusters by using Louvain algorithm and setting resolution parameter at 1.2. Cell clusters were visualized by using UMAP.

### Data Preprocessing and Cluster Identification

Raw reads from RNA‐seq were processed to generate gene expression matrixes using CeleScope (https://github.com/singleron‐RD/CeleScope) v1.9.0 pipeline. Briefly, raw reads were first processed with CeleScope to remove low quality reads with Cutadapt v1.17 to trim poly‐A tail and adapter sequences.^[^ ^36^ ^]^ Cell barcode and UMI were extracted. After that, STAR v2.6.1a was used to map reads to the reference genome GRCm38 (ensemble version 92 annotation).^[^ ^37^ ^]^ UMI counts and gene counts of each cell were acquired using featureCounts v2.0.1 software,^[^ ^38^ ^]^ and used to generate expression matrix files for subsequent analysis.

Cells were filtered using UMI counts of >30 000, gene counts <200 or >5000, and mitochondrial content >20%. After filtering, cells were retained for the downstream analyses. Functions from Seurat v3.1.2 for dimension‐reduction and clustering were used.^[^ ^39^ ^]^ Then NormalizeData and ScaleData functions were used to normalize and scale all gene expressions and the top 2000 variable genes with FindVariableFeautres function were selected for PCA analysis. Using the top 20 principal components, cells were separated into multiple clusters with FindClusters. Batch effect between samples was removed by Harmony.^[^ ^40^ ^]^ Finally, UMAP algorithm was applied to visualize cells in a 2D space.

### DEG

The Seurat FindMarkers function based on Wilcox likelihood‐ratio test with default parameters was used, and the genes expressed in >10% of the cells in a cluster and with an average Log (Fold Change) value >0.25 as DEGs were selected. For the cell type annotation of each cluster, the expression of canonical markers found in the DEGs with knowledge from literatures were combined and displayed the expression of markers of each cell type with heatmaps/dot plots/violin plots that were generated with Seurat function. Doublet cells were identified as expressing markers for different cell types and removed manually.

### Constructing Trajectory in Pseudotime

Cell differentiation trajectory was reconstructed with Monocle2.^[^ ^41^ ^]^ Highly variable genes (HVGs) were used to sort cells in order of spatial‐temporal differentiation. DDRTree was used to perform FindVairableFeatures and dimension‐reduction. Finally, the trajectory was visualized by plot_cell_trajectory function. Next, CytoTRACE (a computational method that predicts the differentiation state of cells from single‐cell RNA‐sequencing data using gene Counts and Expression) was used to predict the differentiation potential of monocyte subpopulations.^[^ ^42^ ^]^

### Pathway Enrichment Analysis

To investigate the potential functions of DEGs, genes were screened that differed significantly (LogFoldchange>0.25, *P* < 0.05) between groups or clusters. Afterward, to investigate the potential functions of these different expression genes, the Gene Ontology (GO) and KEGG analysis were used with the “clusterProfile” R packager.^[^ ^43^ ^]^ Pathways with P_adj value <0.05 were considered as significantly enriched. Gene Ontology gene sets including molecular function (MF), biological process (BP), and cellular component (CC) categories were used as reference.

GSEA was performed on genes in cell clusters. For GSVA pathway enrichment analysis, the average gene expression of each cell type was used as input data using the GSVA package.^[^ ^44^ ^]^

Protein‐protein interactions (PPI) of DEGs in each cluster were predicted based on known interactions of genes with relevant GO terms in the StringDB v1.22.0.

### Pathological and Electron Microscopy Analysis

After cardiac perfusion, the hearts were removed quickly from the mice, wiped gently with a clean absorbent paper and cut horizontally in half. Half of the heart tissues were taken using paraformaldehyde (V900894, Merck), and the remaining tissues were stored at −80 °C.

Hearts were dehydrated and subsequently paraffin‐embedded and then sectioned at 4 µm thickness for pathological analysis. Hematoxylin‐eosin (HE), Masson's trichrome (ab150686, Abcam), TUNEL (C1091, C1089, Beyotime; 11 684 795 910, Roche), Sirius Red (S8060, Solarbio), and WGA (1:200, W834, Thermo) staining were performed according to standard protocols. The slides were analyzed using an automatic digital slide‐scanning system (Axio Scan Z1, Zeiss). For electron microscopy, hearts were fixed in 4% paraformaldehyde, 1.5% glutaraldehyde in 0.1 mol L^−1^ Cacodylate buffer. The hearts were subjected to immersion fixation in the same fixative and embedded in epoxy resin following standard protocols.

### IHC

The heart sections were repaired with antigen, blocked, and incubated with primary antibody overnight at 4 °C (1:500, Rabbit Anti‐PICALM antibody, bs‐11665R, Bioss). The secondary antibody (PV‐6000, Zsgb Bio) conjugated to horseradish peroxidase (HRP) was then incubated at room temperature for 1 h. A DAB kit (ZLI‐9019, Zsgb Bio) was used according to the manufacturer's instructions. Whole slides were scanned with an automatic digital slide‐scanning system (Axio Scan Z1, Zeiss). The IHC analysis was used to detect PICALM protein expression in the heart tissue.

### OMIHC

To analyze the correlation between Aβ and PICALM was observed in situ, the following protocol was followed: step 1: slide preparation and dewaxing; step 2: microwave treatment for epitope retrieval; step3: blocking; step 4: primary antibody incubation (1:500 anti‐PICALM, bs‐11665R, Bioss; 1:1000 anti‐TNNT2, bs‐10648R, Bioss; 1:500 anti‐ Aβ1‐40, bs‐0106 M, Bioss; 1:500 anti‐Aβ1‐42, bs‐0107R, Bioss); step 5: introduction of secondary HRP conjugated secondary antibody; step 6: incubation of opal fluorophore; step 7: microwave treatment for antibody stripping; step 8: using DAPI (1:300) and WGA (1:200) for staining. Steps 3–7 were repeated until all targets of interest were detected using different Opal fluorophores (DAPI‐405, WGA‐488, PICALM‐540, TNNT2‐620, Aβ1‐40‐570, Aβ1‐42‐650). The slides were analyzed using the Vectra Quantitative 14 Pathology Imaging System (PerkinElmer).

### ELISA

To detect trace levels of Aβ protein in the samples, a more sensitive ELISA kit (E‐EL‐M3009/E‐EL‐H0542c, Elabscience) was selected to determine Aβ1‐40 in mice, human, and hiPSC‐CMs. Duplicate readings were averaged for each standard and sample, and then the average zero standard optical density was subtracted. A four‐parameter Logistic curve was plotted on the log‐log axis, with standard concentration on the x‐axis and OD values on the y‐axis.

### Transfection of Cells with siRNAs and Plasmids

The expression of PICALM was depleted using siRNA as previously described.^[^ ^45^ ^]^ Briefly, cells were seeded in a 6 well plate at a density of 150 000 cells well^−1^ 24 h before treatment. All procedures were performed in Opti‐MEM in without serum. Complexes were pre‐formed between the oligonucleotides and oligofectamine (Invitrogen) using 50 pmol oligonucleotide per 4 µl oligofectamine in a total volume of 200 µl Opti‐MEM. The complex was subsequently added to cells to give a final oligonucleotide concentration of 50 nm in a total of 1 mL Opti‐MEM. Cells were incubated with media, siRNA for green fluorescent protein (GFP) to control for off‐target effects of oligofectamine and oligonucleotide. 500 µl of Opti‐MEM supplemented with 12% (v/v) FBS were then added directly to the transfection mixture and cells were then incubated for 48 h and processed as required. The expression of PICALM was overexpressed by adding the amount of CALM‐pmCherry‐N1 plasmid (27 691, addgene) that was twice that of normal protein expression during transfection.

### Immunofluorescence (IF) Analysis

To determine the localization and expression of protein in cardiac tissue, an IF analysis of heart section was performed. After antigen repair, closure, and permeability, the primary antibody (anti‐PICALM; anti‐TNNT; anti‐LPIN1, bs‐0759R, bioss; anti‐APP, bs‐0112 M, bioss; anti‐Aβ40) incubation, sections were incubated with a secondary antibody with Alexa Fluor Dye (Thermo Fisher Scientific). Finally, DAPI and WGA (L4895, Sigma, Shanghai, China) staining was used to express the nuclei and cell membranes.

### Cell Counting Kit‐8 (CCK‐8) Assay

Cells inoculated in a 96‐well plate were incubated with DOX at different time points, and then left untreated or treated with si‐PICALM for the next day. Then, CCK‐8 reagent (CCK8‐500, Hanbio, Shanghai, China) was added to the cells in the dark. After incubation at 37 °C for 3 h, the plate was detected at 450 nm using a microplate reader.

### IncuCyte Imaging Analysis

H9c2 cells pre‐stained with 5 µm carboxyfluorescein succinimidyl ester (CFSE, HY‐D0938, MCE)/10 µm IncuCyte CytoLight Rapid Red Reagent (4706, Sartorius) for 30 min were seed at ≈70% density into a 96‐well plate in triplicate. H9c2 cells pre‐stained with 0.5 µm LysoTracker Red (HY‐D1300, MCE) for 30 min were seeded at ≈70% density in a 96‐well plate in triplicate. The cells of the plates were treated with 1 µm DOX and placed into the IncuCyte imaging system (IncuCyte S3) for 24 h.

### Western Blot (WB)

RIPA lysis buffer (P0013B, Beyotime) and Cocktail (4 693 132 001, Roche) were added to the samples, which were then homogenized on ice. The supernatant was removed and denatured using a protein loading buffer after centrifugation. The protein was transferred to a nitrocellulose membrane (IB23001, Thermo Fisher) after electrophoresis, blocked with skim milk powder, and incubated with the following primary antibodies overnight: anti‐APP (ab32136, Abcam), anti‐PICALM, anti‐Aβ1‐40 (ab254345, Abcam), and anti‐Aβ1‐42. The next day, the corresponding secondary antibody was used for continuous incubation, and the images were collected and processed after exposure to enhanced chemiluminescence (ECL), GAPDH was used as an internal control. ECL‐plus reagent (GE Healthcare) was used to analyze the protein expression level.

### Hochest, PI, Rhod‐4, FITC‐Phalloidin, 2,7‐Dichlorodihydrofluorescein Diacetate (DCFH‐DA), and FITC‐Dextran/DiI ‐LDL Staining

To visually display the vesicle size, α‐actin, living cells, dead cells, ROS, Ca^2+^, and cell permeability, 20 µg mL^−1^ WGA (L4895, Sigma, Shanghai, China), 20 µg mL^−1^ FITC‐phalloidin(ab235137, Abcam, Shanghai, China), 20 µg mL^−1^ Hochest 33 342 (875756‐97‐1, MERCK), 10 µg mL^−1^ PI (HY‐D0815, MCE), 5 µm DCFH‐DA (D6883, Sigma, Shanghai, China), 5 µM Rhod‐4 (T0404, Warbio, Nanjing, China), and 0.1 mg mL^−1^ FITC‐Dextran (HY‐128868D, MCE)/40 µg mL^−1^ DiI‐LDL(20614ES76, Yeasen) were used to stain for 30 min in accordance with the instructions and then washed with PBS.

### TCGA Data Acquisition and Survival Analysis

Bulk RNA‐seq transcriptome data and clinical information were downloaded from TCGA database (https://portal.gdc.cancer.gov/). Simultaneously, the clinical information and follow‐up data of the patients were also downloaded. Before conducting the survival analysis, the expression level of PICALM was extracted and used to rank the patients in descending order. Subsequently, the patients were categorized into two groups based on the median value of PICALM expression: a high PICALM expression group and a low PICALM expression group. Finally, differences in overall survival and progression‐free interval between high and low groups were compared using Kaplan‐Meier curves, with *P*‐values calculated via Log‐rank test, using the Survival package in R.

### Statistical Analysis

The images and WB results were analyzed using ImageJ software. Protein expression by IHC was determined using the plugin of the IHC Toolbox in ImageJ. GraphPad software was used for statistical analysis. All continuous variables are described as the mean ± SD. Student's t‐test was used to analyze the differences between two groups. Multiple‐group comparisons were performed using a by one‐way analysis of variance followed by the Tukey's test. P < 0.05 indicated a statistically significant difference. ^*^ *P* < 0.05; ^**^ *P* < 0.01; ^***^ *P* < 0.001.

## Conflict of Interest

The authors declare no conflict of interest.

## Author Contributions

M.B, X.H., X.C., and T.A. contributed equally to this work. M.B., X.H., and J.S. performed conceptualization; X.C. and T.A. acquired resources; M.B. and X.H. wrote the original draft; H.M., Z.S., M.T., and G.Y. wrote, reviewed, and edited the manuscript; X.H. and J.S. acquired funding; and J.S. performed supervision.

## Supporting information

Supporting Information

Supplemental Table 1

Supplemental Table 2

Supplemental Table 3

## Data Availability

The data that support the findings of this study are available from the corresponding author upon reasonable request.

## References

[1] D. Cardinale , A. Colombo , G. Bacchiani , I. Tedeschi , C. A. Meroni , F. Veglia , M. Civelli , G. Lamantia , N. Colombo , G. Curigliano , C. Fiorentini , C. M. Cipolla , Circulation 2015, 131, 1981. 25948538 10.1161/CIRCULATIONAHA.114.013777

[2] C. Galan‐Arriola , R. Villena‐Gutierrez , M. I. Higuero‐Verdejo , I. A. Diaz‐Rengifo , G. Pizarro , G. J. Lopez , A. Molina‐Iracheta , C. Perez‐Martinez , R. D. Garcia , D. Gonzalez‐Calle , M. Lobo , P. L. Sanchez , E. Oliver , R. Cordoba , V. Fuster , J. Sanchez‐Gonzalez , B. Ibanez , Cardiovasc. Res. 2021, 117, 1132. 32597960 10.1093/cvr/cvaa181 PMC7983009

[3] B. Kalyanaraman , Redox Biol. 2020, 29, 101394. 31790851 10.1016/j.redox.2019.101394 PMC6909145

[4] L. Wu , L. Wang , Y. Du , Y. Zhang , J. Ren , Trends Pharmacol. Sci. 2022, 44, 34. 36396497 10.1016/j.tips.2022.10.003

[5] A. Arinno , C. Maneechote , T. Khuanjing , B. Ongnok , N. Prathumsap , T. Chunchai , B. Arunsak , S. Kerdphoo , K. Shinlapawittayatorn , S. C. Chattipakorn , N. Chattipakorn , Biochem. Pharmacol. 2021, 192, 114743. 34453902 10.1016/j.bcp.2021.114743

[6] a) P. H. Reddy , M. F. Beal , Trends Mol. Med. 2008, 14, 45; 18218341 10.1016/j.molmed.2007.12.002 PMC3107703

[7] a) S. Greco , G. Zaccagnini , P. Fuschi , C. Voellenkle , M. Carrara , I. Sadeghi , C. Bearzi , B. Maimone , S. Castelvecchio , K. Stellos , C. Gaetano , L. Menicanti , F. Martelli , Cardiovasc. Res. 2017, 113, 453; 28158647 10.1093/cvr/cvx013

[8] M. H. Dreyling , J. A. Martinez‐Climent , M. Zheng , J. Mao , J. D. Rowley , S. K. Bohlander , Proc. Natl. Acad. Sci. USA 1996, 93, 4804. 8643484 10.1073/pnas.93.10.4804 PMC39360

[9] a) M. G. Ford , B. M. Pearse , M. K. Higgins , Y. Vallis , D. J. Owen , A. Gibson , C. R. Hopkins , P. R. Evans , H. T. McMahon , Science 2001, 291, 1051; 11161218 10.1126/science.291.5506.1051

[10] L. Troncone , M. Luciani , M. Coggins , E. H. Wilker , C. Y. Ho , K. E. Codispoti , M. P. Frosch , R. Kayed , F. Del Monte , J. Am. Coll. Cardiol. 2016, 68, 2395. 27908343 10.1016/j.jacc.2016.08.073 PMC5142757

[11] R. B. Mukku , G. C. Fonarow , K. E. Watson , O. A. Ajijola , E. C. Depasquale , A. Nsair , A. S. Baas , M. C. Deng , E. H. Yang , J. Card. Fail. 2016, 22, 439. 27109619 10.1016/j.cardfail.2016.04.009

[12] M. Li , V. Sala , M. C. De Santis , J. Cimino , P. Cappello , N. Pianca , A. Di Bona , J. P. Margaria , M. Martini , E. Lazzarini , F. Pirozzi , L. Rossi , I. Franco , J. Bornbaum , J. Heger , S. Rohrbach , A. Perino , C. G. Tocchetti , B. H. F. Lima , M. M. Teixeira , P. E. Porporato , R. Schulz , A. Angelini , M. Sandri , P. Ameri , S. Sciarretta , R. C. P. Lima‐Junior , M. Mongillo , T. Zaglia , F. Morello , et al., Circulation 2018, 138, 696. 29348263 10.1161/CIRCULATIONAHA.117.030352

[13] C. Basso , E. Czarnowska , M. D. Barbera , B. Bauce , G. Beffagna , E. K. Wlodarska , K. Pilichou , A. Ramondo , A. Lorenzon , O. Wozniek , D. Corrado , L. Daliento , G. A. Danieli , M. Valente , A. Nava , G. Thiene , A. Rampazzo , Eur. Heart J. 2006, 27, 1847. 16774985 10.1093/eurheartj/ehl095

[14] W. Luo , X. Zou , Y. Wang , Z. Dong , X. Weng , Z. Pei , S. Song , Y. Zhao , Z. Wei , R. Gao , B. Zhang , L. Liu , P. Bai , J. Liu , X. Wang , T. Gao , Y. Zhang , X. Sun , H. Chen , K. Hu , S. Du , A. Sun , J. Ge , Circ. Res. 2023, 132, e223. 37154056 10.1161/CIRCRESAHA.122.321587

[15] H. Zhang , X. Hua , J. Song , Phenomics 2021, 1, 229. 36939805 10.1007/s43657-021-00022-1 PMC9590492

[16] a) J. Inserte , D. Garcia‐Dorado , Br. J. Pharmacol. 2015, 172, 1996; 25297462 10.1111/bph.12959 PMC4386977

[17] a) Y. Sun , L. Wu , Y. Zhong , K. Zhou , Y. Hou , Z. Wang , Z. Zhang , J. Xie , C. Wang , D. Chen , Y. Huang , X. Wei , Y. Shi , Z. Zhao , Y. Li , Z. Guo , Q. Yu , L. Xu , G. Volpe , S. Qiu , J. Zhou , C. Ward , H. Sun , Y. Yin , X. Xu , X. Wang , M. A. Esteban , H. Yang , J. Wang , M. Dean , et al., Cell 2021, 184, 404; 33357445 10.1016/j.cell.2020.11.041

[18] C. R. Taylor , R. M. Levenson , Histopathology 2006, 49, 411. 16978205 10.1111/j.1365-2559.2006.02513.x

[19] a) U. C. Muller , T. Deller , M. Korte , Nat. Rev. Neurosci. 2017, 18, 281; 28360418 10.1038/nrn.2017.29

[20] a) K. Stamatelopoulos , C. J. Pol , C. Ayers , G. Georgiopoulos , A. Gatsiou , E. S. Brilakis , A. Khera , K. Drosatos , J. A. de Lemos , K. Stellos , J. Am. Coll. Cardiol. 2018, 72, 1060; 30139434 10.1016/j.jacc.2018.06.027 PMC6467498

[21] K. Piorecka , A. Janaszewska , M. Majkowska , M. Marcinkowska , J. Kurjata , S. Kazmierski , E. Radzikowska‐Cieciura , B. Kost , P. Sowinski , B. Klajnert‐Maculewicz , W. A. Stanczyk , Materials 2020, 13, 5512. 33287168 10.3390/ma13235512 PMC7730793

[22] K. Fujimaki , R. Li , H. Chen , K. Della Croce , H. H. Zhang , J. Xing , F. Bai , G. Yao , Biol. Sci. 2019, 116, 22624. 10.1073/pnas.1915905116 PMC6842626 31636214

[23] L. Fassina , G. Magenes , A. Inzaghi , S. Palumbo , G. Allavena , C. Miracco , L. Pirtoli , M. Biggiogera , S. Comincini , Eur. J. Histochem. : EJH 2012, 56, 44. 10.4081/ejh.2012.e44 PMC3567763 23361240

[24] a) H. Abdel‐Qadir , P. C. Austin , D. S. Lee , E. Amir , J. V. Tu , P. Thavendiranathan , K. Fung , G. M. Anderson , JAMA Cardiol. 2017, 2, 88; 27732702 10.1001/jamacardio.2016.3841

[25] T. Abdul‐Rahman , A. Dunham , H. Huang , S. M. A. Bukhari , A. Mehta , W. A. Awuah , D. Ede‐Imafidon , E. Cantu‐Herrera , S. Talukder , A. Joshi , D. W. Sundlof , R. Gupta , Curr. Probl. Cardiol. 2023, 48, 101591. 36621516 10.1016/j.cpcardiol.2023.101591

[26] G. F. Chen , T. H. Xu , Y. Yan , Y. R. Zhou , Y. Jiang , K. Melcher , H. E. Xu , Acta Pharmacol. Sin. 2017, 38, 1205. 28713158 10.1038/aps.2017.28 PMC5589967

[27] T. A. Kokjohn , G. D. Van Vickle , C. L. Maarouf , W. M. Kalback , J. M. Hunter , I. D. Daugs , D. C. Luehrs , J. Lopez , D. Brune , L. I. Sue , T. G. Beach , E. M. Castano , A. E. Roher , Biochim. Biophys. Acta 2011, 1812, 1508. 21784149 10.1016/j.bbadis.2011.07.004 PMC3185199

[28] H. E. Mohamad , D. M. Abo‐Elmatty , N. S. Wahba , M. A. Shaheen , R. T. Sakr , A. S. Wahba , Life Sci. 2022, 301, 120613. 35523286 10.1016/j.lfs.2022.120613

[29] J. H. Viles , Angew. Chem., Int. Ed. Engl. 2023, 62, 202215785.

[30] L. G. Hall , J. K. Czeczor , T. Connor , J. Botella , K. A. De Jong , M. C. Renton , A. J. Genders , K. Venardos , S. D. Martin , S. T. Bond , K. Aston‐Mourney , K. F. Howlett , J. A. Campbell , G. R. Collier , K. R. Walder , M. McKenzie , M. Ziemann , S. L. McGee , Nat. Commun. 2024, 15, 258. 38225272 10.1038/s41467-023-44520-4 PMC10789867

[31] J. W. Lovelace , V. Augustine , Nature 2023, 10.1038/d41586-023-03076-5.

[32] a) X. Lian , C. Hsiao , G. Wilson , K. Zhu , L. B. Hazeltine , S. M. Azarin , K. K. Raval , J. Zhang , T. J. Kamp , S. P. Palecek , Proc. Natl. Acad. Sci. USA 2012, 109, E1848; 22645348 10.1073/pnas.1200250109 PMC3390875

[33] X. Hua , G. Hu , Q. Hu , Y. Chang , Y. Hu , L. Gao , X. Chen , P. C. Yang , Y. Zhang , M. Li , J. Song , Circulation 2020, 142, 384. 32431172 10.1161/CIRCULATIONAHA.119.043545

[34] L. Chen , K. Hua , N. Zhang , J. Wang , J. Meng , Z. Hu , H. Gao , F. Li , Y. Chen , J. Ren , L. Liu , Q. Zhou , J. Gu , J. Song , X. Zhang , S. Hu , Circulation 2022, 145, 315. 35073179 10.1161/CIRCULATIONAHA.121.055690

[35] F. A. Wolf , P. Angerer , F. J. Theis , Genome Biol. 2018, 19, 15. 29409532 10.1186/s13059-017-1382-0 PMC5802054

[36] M. Martin , EMBnet. J. 2011, 17, 10.

[37] A. Dobin , C. A. Davis , F. Schlesinger , J. Drenkow , C. Zaleski , S. Jha , P. Batut , M. Chaisson , T. R. Gingeras , Bioinformatics 2013, 29, 15. 23104886 10.1093/bioinformatics/bts635 PMC3530905

[38] Y. Liao , G. K. Smyth , W. Shi , Bioinformatics 2014, 30, 923. 24227677 10.1093/bioinformatics/btt656

[39] R. Satija , J. A. Farrell , D. Gennert , A. F. Schier , A. Regev , Nat. Biotechnol. 2015, 33, 495. 25867923 10.1038/nbt.3192 PMC4430369

[40] I. Korsunsky , N. Millard , J. Fan , K. Slowikowski , F. Zhang , K. Wei , Y. Baglaenko , M. Brenner , P. R. Loh , S. Raychaudhuri , Nat. Methods 2019, 16, 1289. 31740819 10.1038/s41592-019-0619-0 PMC6884693

[41] X. Qiu , A. Hill , J. Packer , D. Lin , Y. A. Ma , C. Trapnell , Nat. Methods 2017, 14, 309. 28114287 10.1038/nmeth.4150 PMC5330805

[42] G. S. Gulati , S. S. Sikandar , D. J. Wesche , A. Manjunath , A. Bharadwaj , M. J. Berger , F. Ilagan , A. H. Kuo , R. W. Hsieh , S. Cai , M. Zabala , F. A. Scheeren , N. A. Lobo , D. Qian , F. B. Yu , F. M. Dirbas , M. F. Clarke , A. M. Newman , Science 2020, 367, 405. 31974247 10.1126/science.aax0249 PMC7694873

[43] G. Yu , L. G. Wang , Y. Han , Q. Y. He , OMICS 2012, 16, 284. 22455463 10.1089/omi.2011.0118 PMC3339379

[44] S. Hänzelmann , R. Castelo , J. Guinney , BMC Bioinform. 2013, 14, 7. 10.1186/1471-2105-14-7 PMC3618321 23323831

[45] a) R. S. Thomas , A. Henson , A. Gerrish , L. Jones , J. Williams , E. J. Kidd , BMC Neurosci. 2016, 17, 50; 27430330 10.1186/s12868-016-0288-1 PMC4949774

