# Supplementary material for: PICALM Regulating the Generation of Amyloid β‐Peptide to Promote Anthracycline‐Induced Cardiotoxicity
Source: Adv Sci (Weinh). 2024 Jun 27;11(32):2401945. doi: 10.1002/advs.202401945 (PMC11348153; doi:10.1002/advs.202401945)
Supplement: Supplementary file 1 — Supporting Information [file ADVS-11-2401945-s003.docx]

**Supplementary information**

***PICALM Regulating the Generation of Amyloid β-peptide to Promote Anthracycline-induced Cardiotoxicity***

*Mengni Bao, Xiumeng Hua,* *Xiao Chen, Tao An, Han Mo, Zhe Sun, Menghao Tao, Guangxin Yue, Jiangping Song**


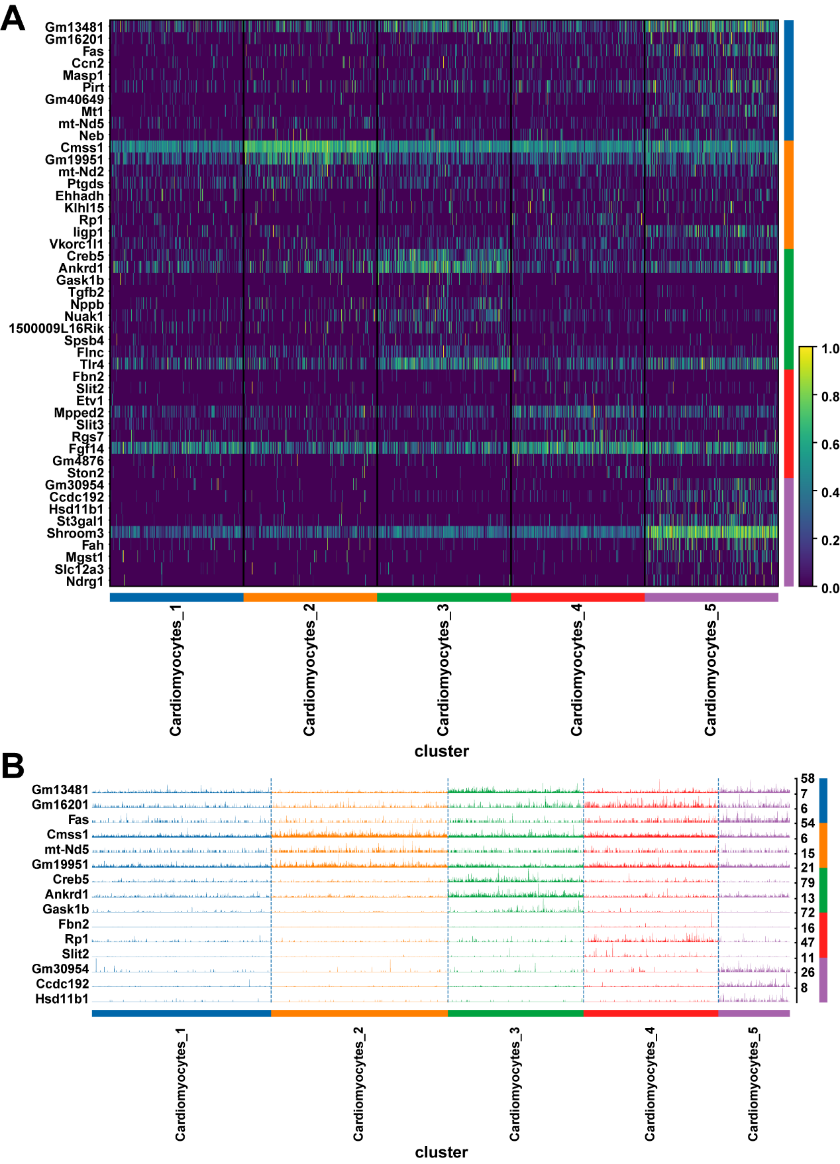


**Figure S1.** Distinct expression of the cell-type-specific genes overlaid on the UMAP of Figure 2A.

**
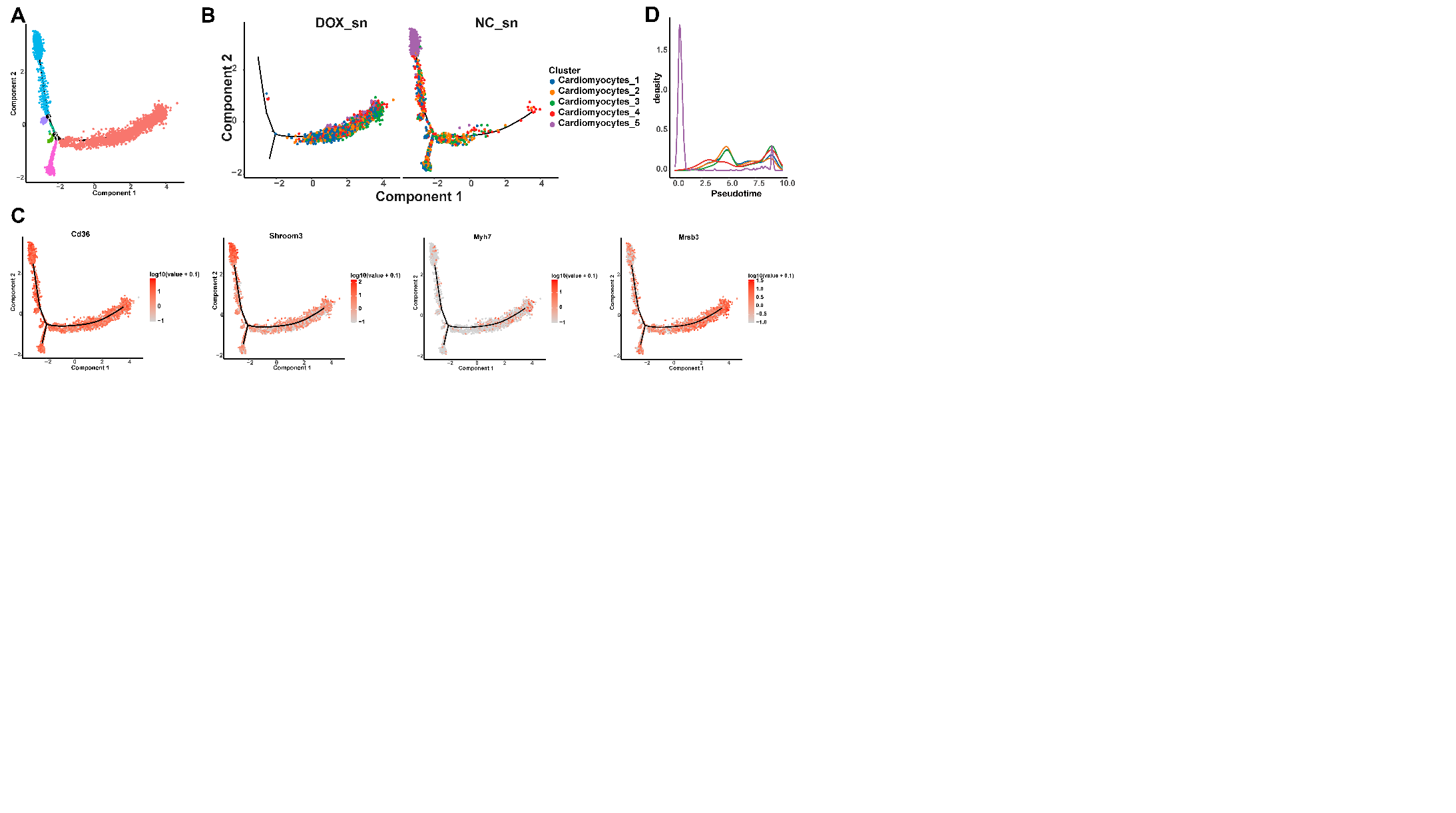
**

**Figure S2.** Pseudo-time analysis of cardiomyocytes in RNA sequencing. A) Pseudo-time analysis of cardiomyocytes in different states. B) Diagram showing the distribution of each sample in the pseudo-temporal trajectory. C) Monocle dimension reduction (DDTree) diagram showing the expression of some selected genes. D) Diagram showing the density distribution of different cell types with pseudo-time.


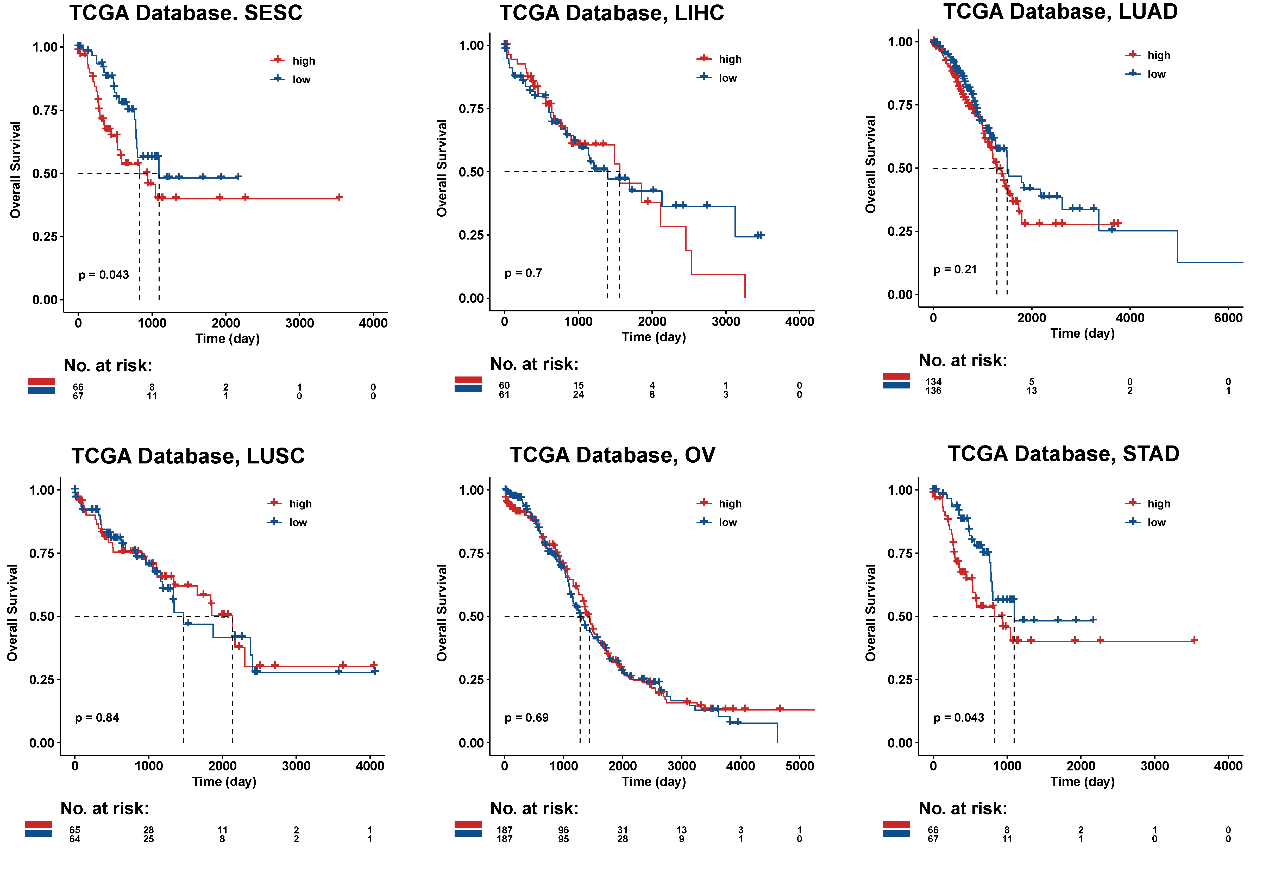


**Figure S3.** The cervical cancer (CESC), liver cancer (LIHC), lung adenocarcinoma (LUAD), lung squamous cell carcinoma (LUSC), ovarian cancer (OV), and stomach cancer (STAD) survival analysis in TCGA database.


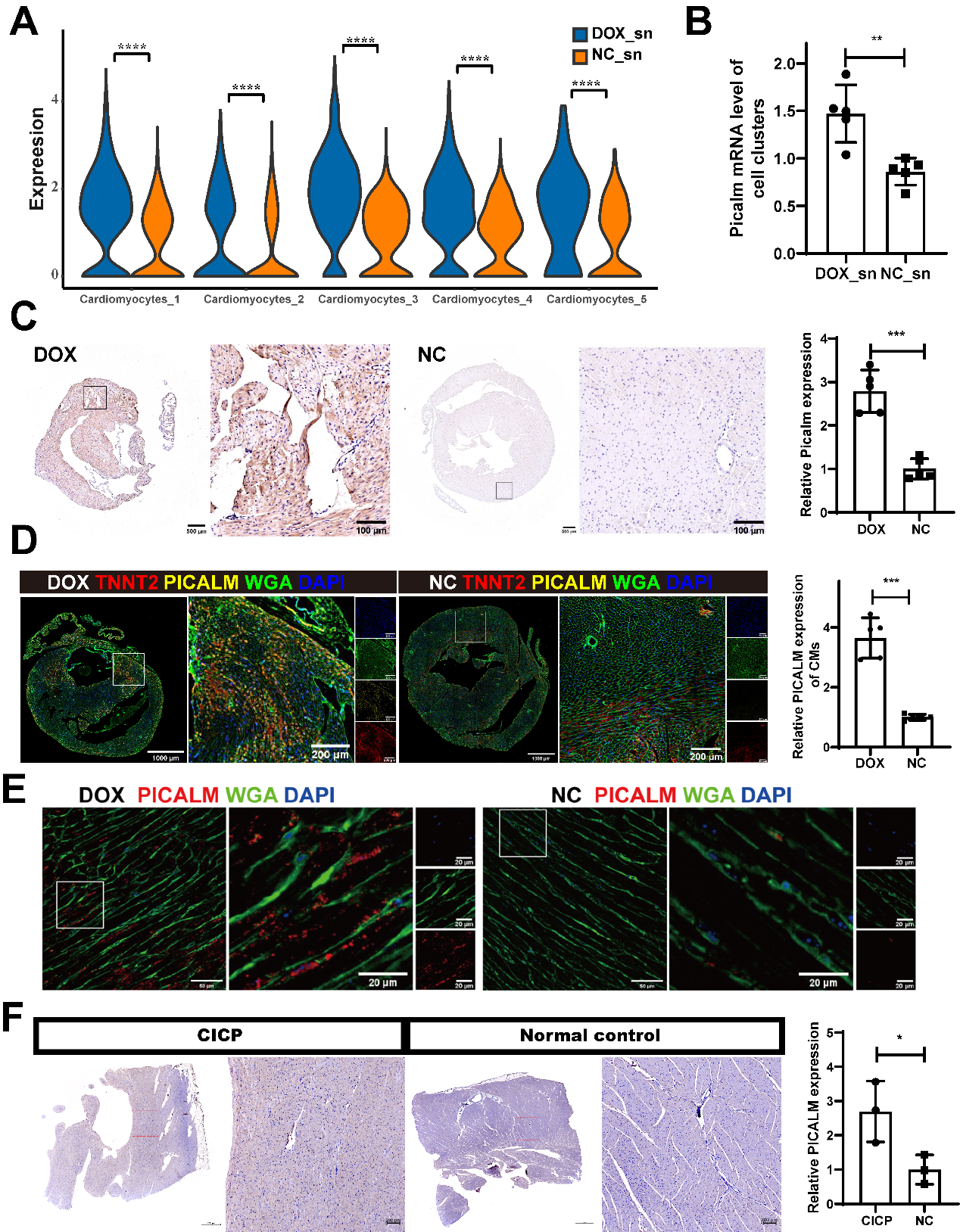


**Figure S4.** PICALM expression in DOX-induced cardiotoxicity model and patients. A) Violin plots showing the expression of *Picalm* gene in each cardiomyocyte cluster. B) Statistical plots showing the difference in *picalm* mRNA level between NC and DOX groups. C) Immunohistochemistry (IHC) representative and statistical graphs of PICALM expression in DOX-treated mice at the tissue level. Scale bars, 500 µm; scale bars for the magnified images, 100 µm. D) Opal multicolor IHC (OMIHC) representative and statistical graphs of PICALM expression at the myocardial cells in DOX-treated mice at the tissue level. Scale bars, 1000 µm; scale bars for the magnified images, 200 µm. E) Immunofluorescence showed *Picalm* expression in cytoplasm and closed to membrane in mice. Scale bars, 50 µm; scale bars for the magnified images, 20 µm. F) IHC representative and statistical graphs of PICALM expression in DOX-induced cardiotoxicity patients at the tissue level. Scale bars, 2000 µm; scale bars for the magnified images, 200 µm. NC, normal control (PBS-treated mice); DOX, doxorubicin-treated mice; CICP, chemotherapy-induced cardiotoxicity patients. Mice: n = 5/group; human: n = 3/group. Statistical analyses were performed using an unpaired t-test. **P* < 0.05; ***P* < 0.01; ****P* < 0.001. Data are presented as the mean ± SD.


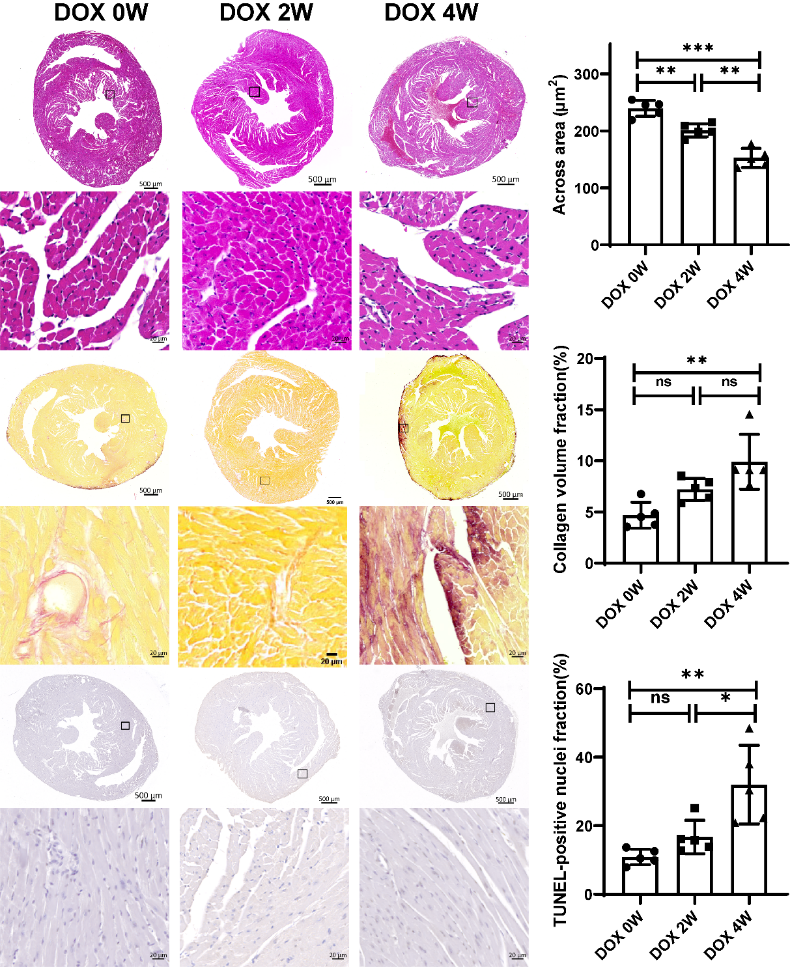


**Figure S5**. Pathology of DOX-induced development in mice. Representative and statistical graphs of HE/Sirius red/TUNEL-stained in mice. Scale bars,500 µm; scale bars for the magnified images, 20 µm. Mice: n=5/group. Student’s t -test was applied to analyze the differences between two groups. Multiple-group comparisons were made by one-way ANOVA followed by the Tukey test. **P* < 0.05; ***P* < 0.01; ****P* < 0.001. Data are presented as the mean ± SD.


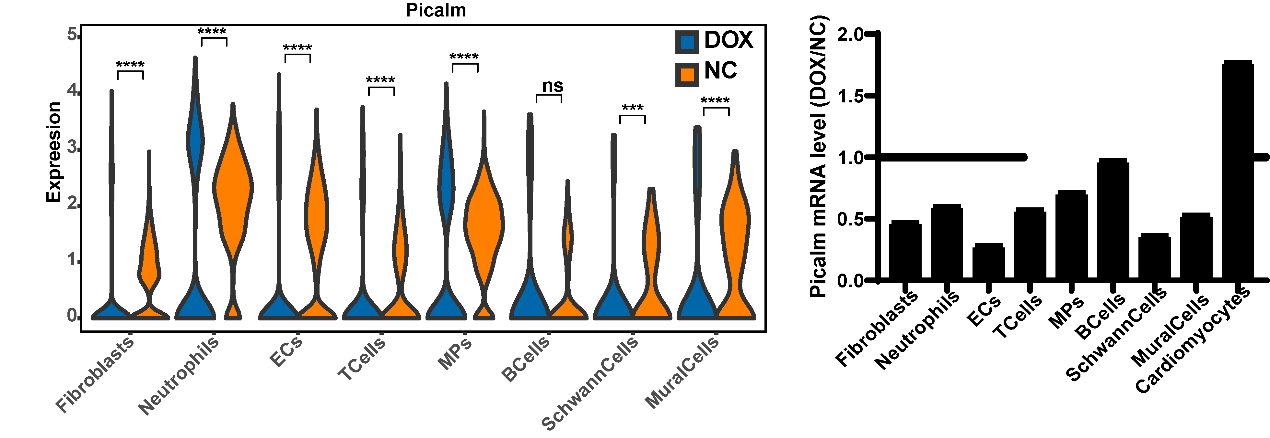


**Figure S6**. Plots showing the expression of Picalm gene in non-cardiomyocyte.


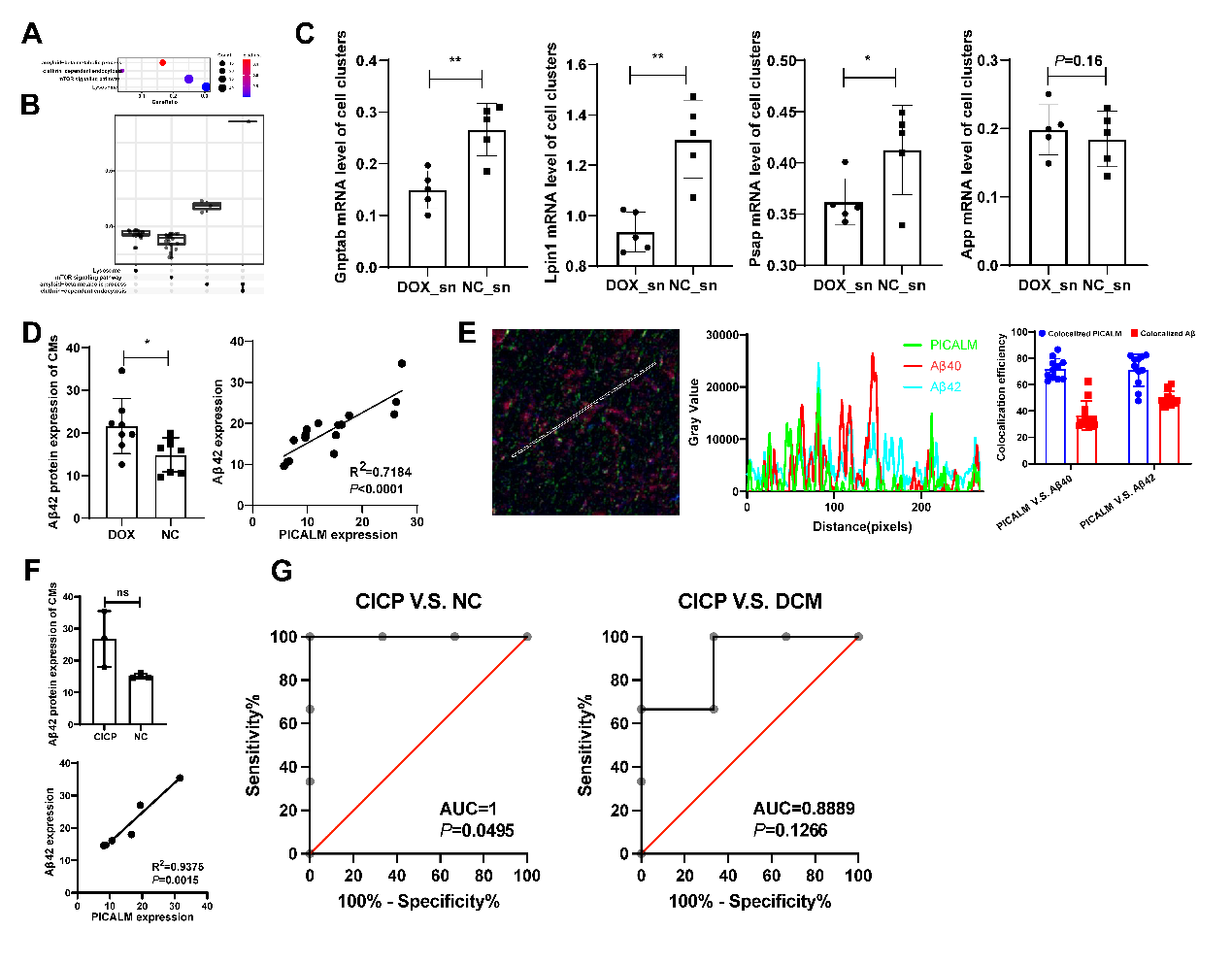


**Figure S7**. Amyloid β metabolic process pathway and related genes were the major issue in DOX-induced cardiotoxicity mice and chemotherapy-induced cardiotoxicity patients. A) Dot plot for GSEA analysis of cardiomyocytes. The color of the circle represents the size of *P*-value, and the size of the circle represents the number of differential genes. B) Diagram for each pathway shared or unique genes. The black dots of the lines between pathways represent common genes of the lines, and the barplot above represents the fold change of unique or common genes. C) Statistical plots showing the difference in Lpin1/Psap/Gnptab/App mRNA level between NC and DOX groups. D) Statistical graphs of Aβ42, Aβ40, PICALM expression at the cardiomyocytes marked by TNNT2 in DOX-induced cardiotoxicity mice and the diagram showing the correlation of PICALM and Aβ42 in mice cardiomyocytes. E) Line profile indicating the distribution of PICALM (green) and Aβ40 (red) /Aβ42 (blue) signals on the line in figure, and histogram of the colocalization efficiency. F) Statistical graphs of Aβ42 expression at the cardiomyocytes in chemotherapy-induced cardiotoxicity patient. And diagram showing the correlation of PICALM and Aβ42 in human cardiomyocytes. G) ROC curve analysis of serum Aβ40 to distinguish the CICP patients. NC, normal control; DOX, doxorubicin-treated mice; CICP, chemotherapy-induced cardiotoxicity patients. Mice: n = 5/group in RNA-seq and n = 8/group in immunoimaging; human: n = 3/group. Statistical analyses were performed using an unpaired t-test. **P* < 0.05; ***P* < 0.01; ****P* < 0.001. Data are presented as the mean ± SD.


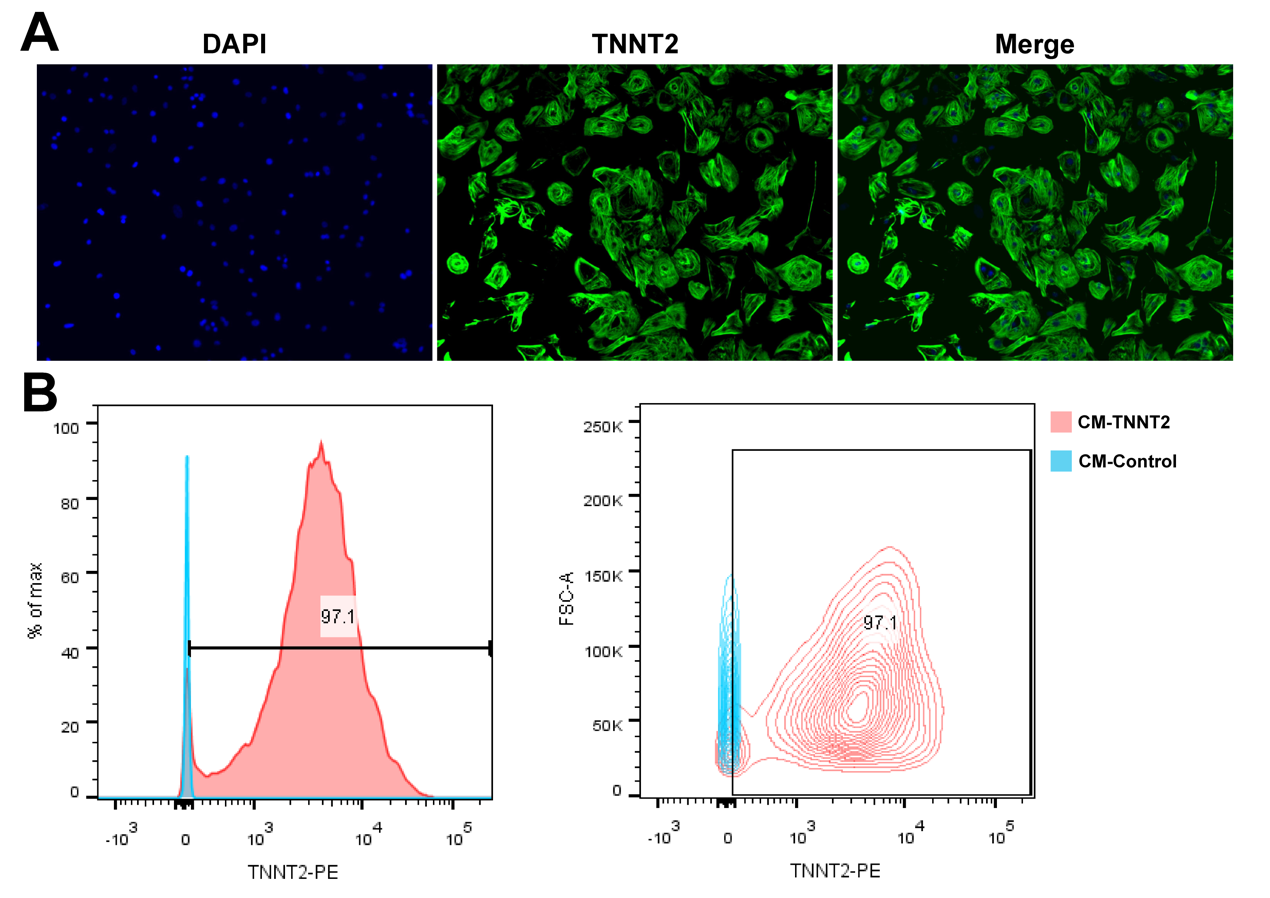


**Figure S8.** hiPSC-CMs used in our study. A) Immunofluorescence of TNNT2 on hiPSC-CMs. B) The flow cytometry analysis the percentage of TNNT2^+^ cells in hiPSC-CMs.


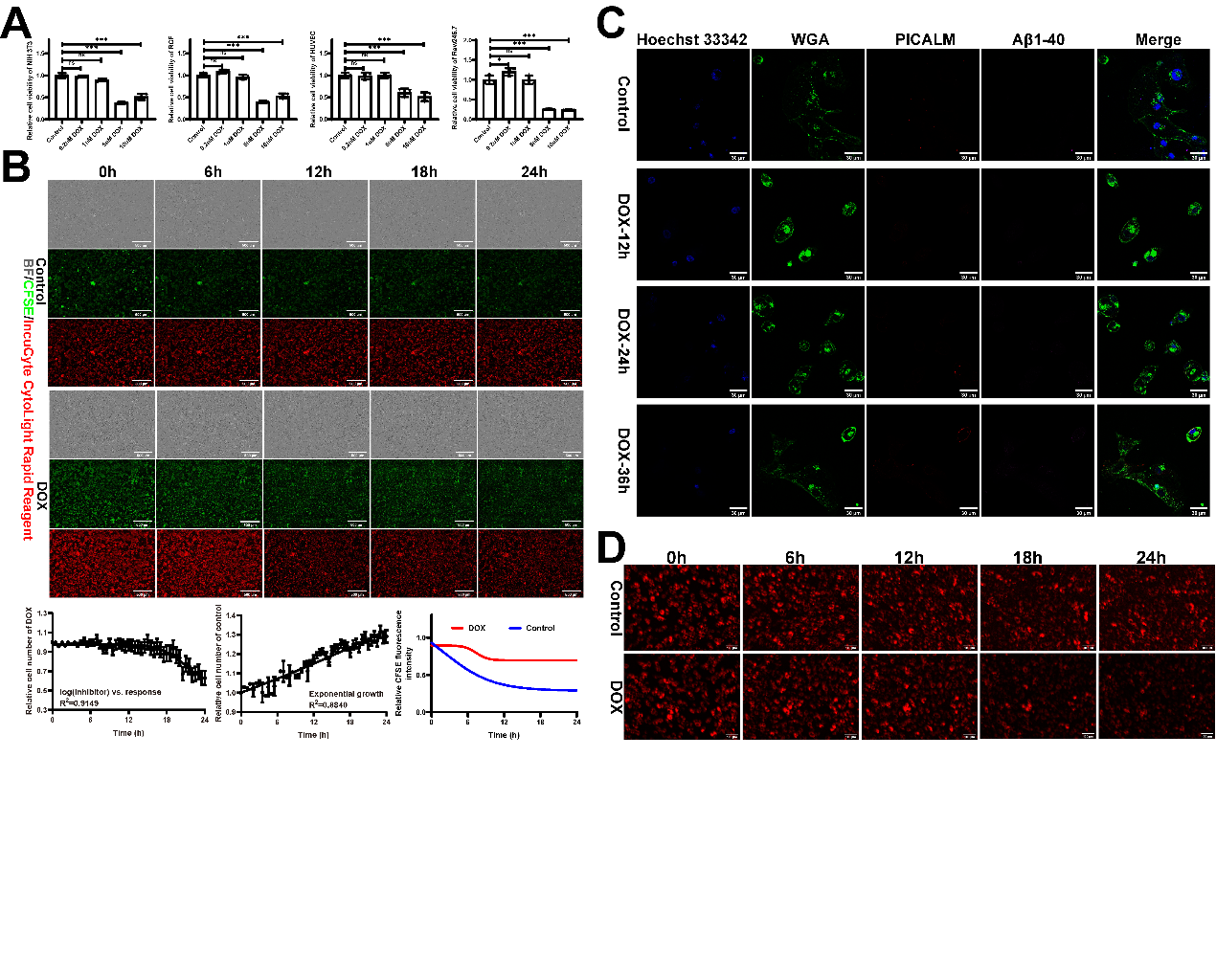


**Figure S9.** DOX-induced H9c2 damage and Aβ peptide production. A) The cell viability in different groups. Cells were treated with the indicated concentration of DOX (0–10 uM) for 24 h. After incubation, cell viability was measured by CCK8 assay. B) The microscopic fluorescence images, and statistical graphs determined by using an IncuCyte imaging system. H9c2 cells pre-stained with 5 μM CFSE/10 uM for 30 min were treated with 1 uM DOX for 24h. C) The microscopic fluorescence images of hiPSC-CMs treated with 1uM DOX for 0h, 12h, 24h and 36h. D) The microscopic fluorescence images and statistical graphs determined using an IncuCyte imaging system. H9c2 cells pre-stained with 0.5μM LysoTracker Red for 30 min were treated with 1 uM DOX for 24h. Student’s t -test was applied to analyze the differences between two groups. Multiple-group comparisons were made by one-way ANOVA followed by the Tukey test. **P* < 0.05; ***P* < 0.01; ****P* < 0.001. Data are presented as the mean ± SD.

**
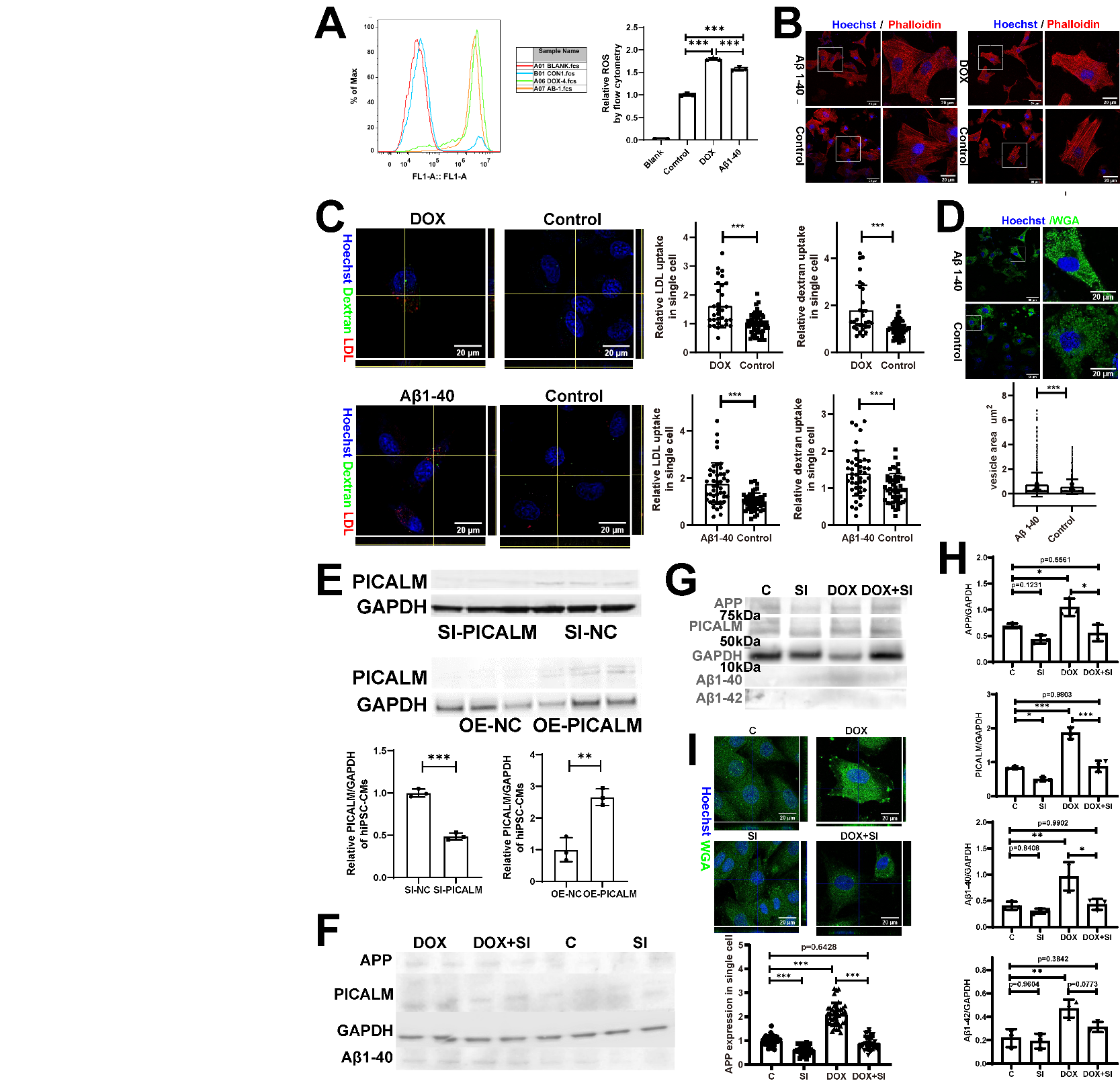
**

**Figure S10.** Si-Picalm can alleviate DOX-induced H9c2 damage and Aβ peptide produce. A) Representative images and quantification of DCFH-DA fluorescence intensity by flow cytometry. B-D) Representative images and quantification of FITC-Dextran/Dil-LDL/rhodamine-Phalloidin/WGA -stained H9c2 cells treated by DOX/ Aβ (1-40) or not. Scale bar, 50 µm. E) The western blot of the groups and statistical graphs of PICALM expression. F-H) The western blot of the groups including C (SI-NC), SI (SI-PICALM), DOX (DOX+SI-NC), DOX+SI (DOX+SI-PICALM), and statistical graphs of Aβ40, Aβ42, PICALM and APP expression. I) The fluorescence images and statistical graphs of APP expression determined by confocal. Fluorescence intensity was quantified by the ImageJ plugin 3D Object Counter. Student’s t -test was applied to analyze the differences between two groups. Multiple-group comparisons were made by one-way ANOVA followed by the Tukey test. **P* < 0.05; ***P* < 0.01; ****P* < 0.001. Data are presented as the mean ± SD.


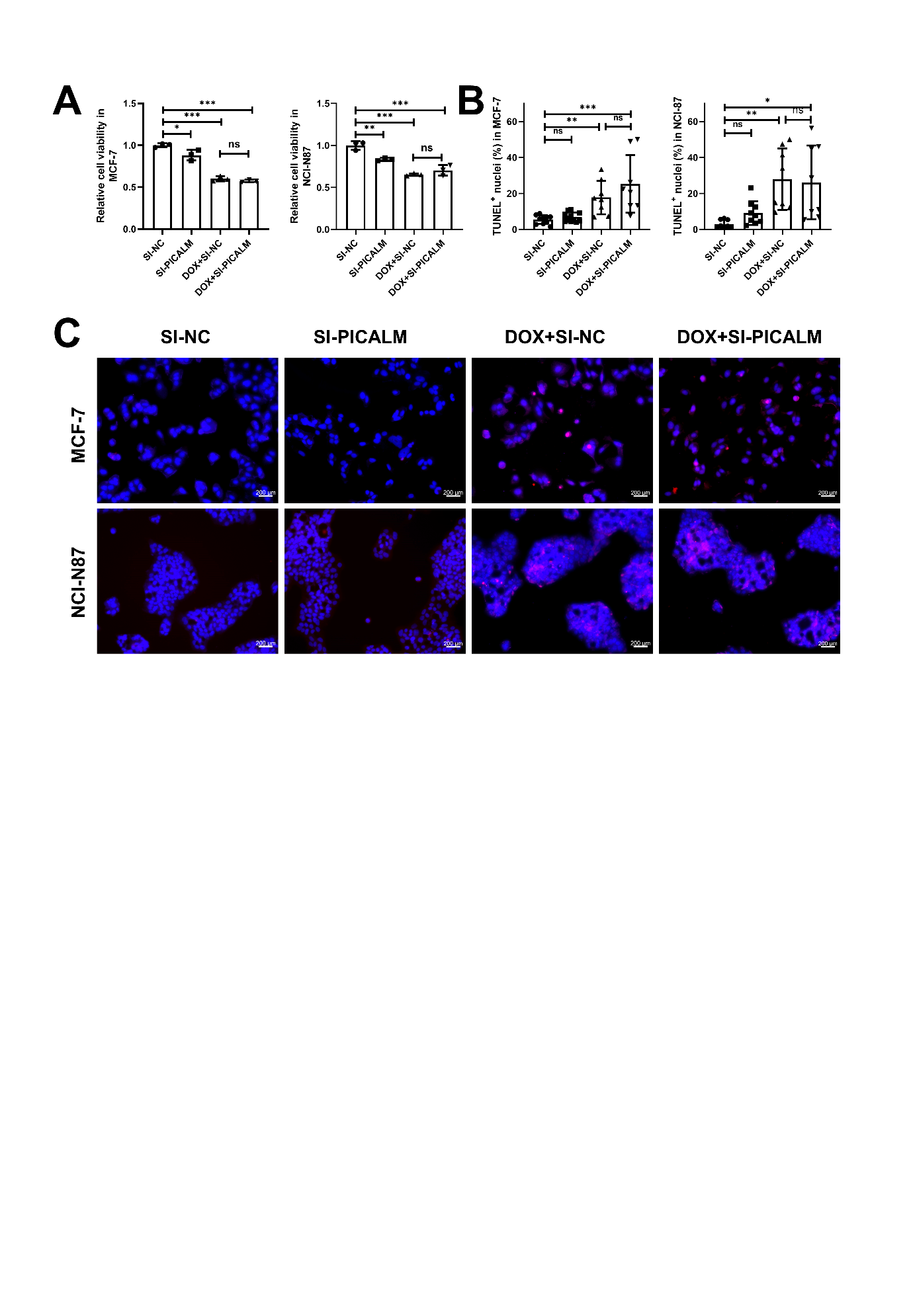


**Figure S11.** The cell viability and the TUNEL assay in groups including SI-NC, SI-PICALM, DOX treatment, DOX treatment + SI-PICALM. A) The cell viability of MCF-7 and NCI-N87 in groups measured by CCK8 assay. B-C) Representative images and quantification of the TUNEL assay. Scale bars, 200 µm. Student’s t -test was applied to analyze the differences between two groups. Multiple-group comparisons were made by one-way ANOVA followed by the Tukey test. **P* < 0.05; ***P* < 0.01; ****P* < 0.001. Data are presented as the mean ± SD.


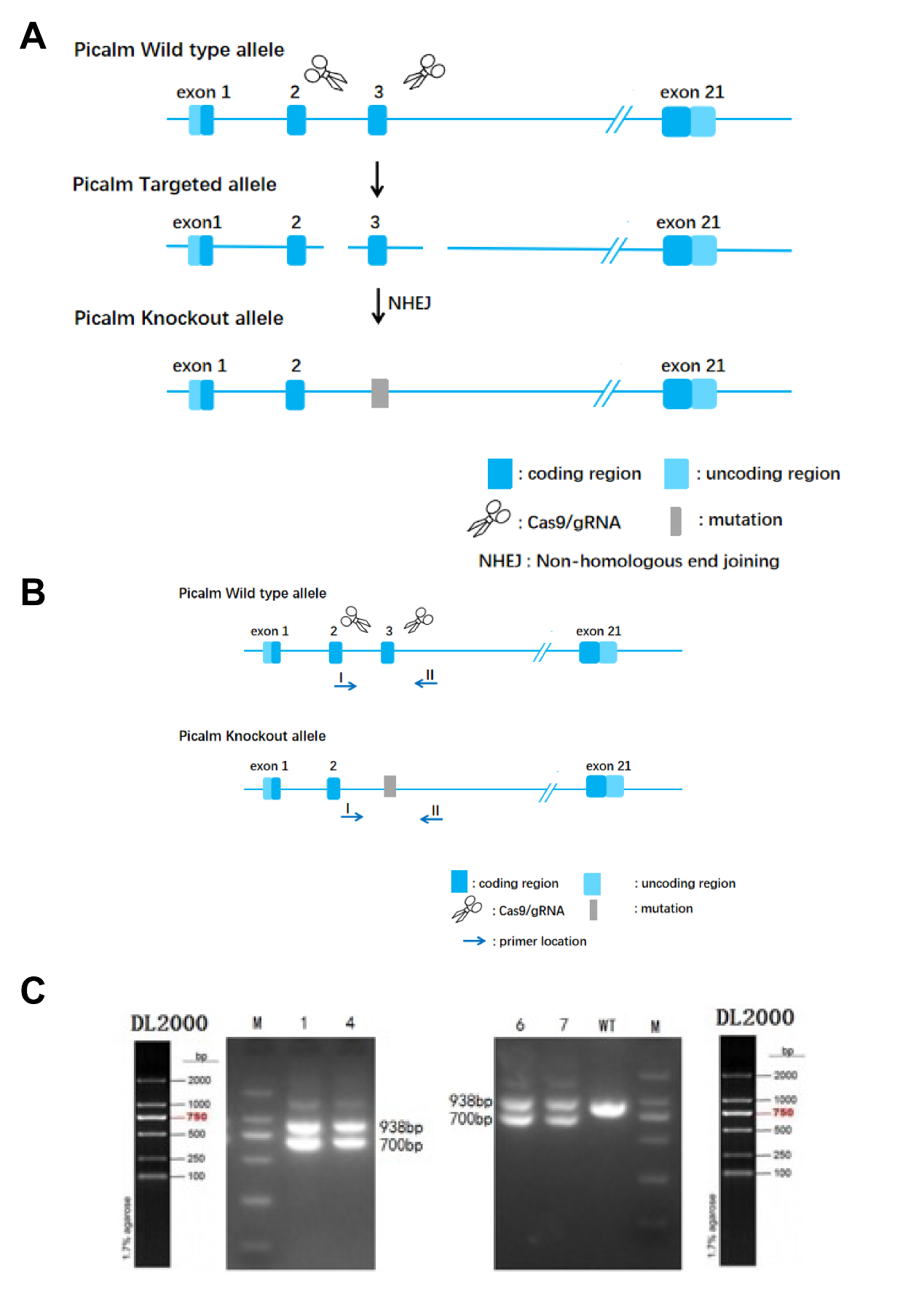


**Figure S12.** PICALM knockout mice. A) Strategy figure to gain Picalm knockout mice. B) Genotyping strategy of F1 mice. C) PCR genotyping of homologous knockout F1 mice.
